# A cross-disciplinary mixed-method approach to understand how food retail environment transformations influence food choice and intake among the urban poor: Experiences from Vietnam

**DOI:** 10.1016/j.appet.2019.104370

**Published:** 2019-11-01

**Authors:** Sigrid C.O. Wertheim-Heck, Jessica E. Raneri

**Affiliations:** a Environmental Policy Group, Department of Social Sciences, Wageningen University, Hollandseweg 1, 6706 KN, Wageningen, the Netherlands; b C/o Food and Healthy Living Group, Aeres University of Applied Sciences, Stadhuisstraat 18, 1315 AK, Almere, the Netherlands; c Healthy Diets from Sustainable Food Systems Initiative, Bioversity International, Via Dei Tre Denari, 472/a, 00054, Maccarese (Fiumicino), Italy

**Keywords:** Nutrition, Social practices, Mixed methods, Consumption, Food safety, Food environment

## Abstract

Nutrition insecurity among urban poor in modernizing Asian metropolises is a critical issue. It is well recognized that in urban Asia the poor are food insecure. Across Asia the food retail environment is transforming rapidly, in which supermarkets increasingly replace traditional food vending, like markets and street vendors that the urban poor depend upon. The question is, how these transformations impact the diets of the urban poor? What drives their food choice? What are their daily shopping practices and how does that affect their dietary intake? To investigate this, we developed a cross-disciplinary nutrition and social practices study with a sequential quantitative-qualitative mixed-method design. Building on empirical evidence from Hanoi, Vietnam, the study links (i) food choice and measured dietary intake, with (ii) food retail environment, through (iii) food shopping practices and preferences of 400 women of reproductive age within the context of (iv) their transformative urban lifestyles. Methods included are a retail census with GPS coordinates to map the food retail environment, a household survey, a 24-h diet recall, multi-generation household interviews and shopping trips. We demonstrate that integrated sociological and nutritional perspectives are productive in rapidly generating evidence to comprehend the complex trade-offs between food safety and nutrition in everyday food consumption practices. We describe and reflect on our theoretical mix of dietary intake and social practices research, and our holistic mixed method approach which besides combining quantitative and qualitative methods, also voices the urban poor first hand.

## 1 Introduction – food retail transformations and the urban poor

Realizing food and nutrition security for the poor is an increasingly tough challenge in urban Asia. More than half the global population currently lives in urban areas, and is expected to grow to 60% in 2030 and 70% by 2050 (UN, 2015). By 2050, 50% of the world's urban population will reside in Asia with the majority being poor (Mathur, 2013; Ravallion, Shaohua, & Prem, 2007; UN, 2014). Among the toughest challenges confronting policy makers is the mounting pressure to ensure adequate, safe and nutritious food intake, and specifically ensuring the food and nutrition security of the urban poor. It is well recognized that in urban Asia the lower income classes are food insecure (Friel & Baker, 2009; Mohiddin, Phelps, & Walters, 2012). Across Southeast Asia (SEA) food retailing systems are transforming rapidly (Moore, 2013; Reardon & Timmer, 2012; 2014; Wrigley & Lowe, 2010) in which modern supermarkets increasingly replace traditional food vending structures such as open markets and street vendors. It is important to uncover how these food environment transformations in SEA impact food intake and dietary quality of the urban poor. Research has demonstrated how the effect of income on dietary consumption is modified by the food environment; elucidating how food and nutrition insecurity may be attributed to food provision interventions (Herforth & Ahmed, 2015; Pingali, 2007). Thus, the future food and nutrition security of the growing number of urban poor depends on food system transformation decisions made now, in preparation for this anticipated urban growth.

### 1.1 Hanoi a case in point

Hanoi, the capital of Vietnam, provides a case in point regarding the food and nutrition security implications of rapid urban population growth and food system transformations. In Vietnam the national urban population is expected to nearly double to 56% in 2050 (43% in 2030). Specifically, the population of Hanoi will grow from about 7.5 million in 2015 to 9 million in 2030 and over 10 million in 2050 (GSO, 2009–2014; UN, 2014). In preparation, Hanoi authorities developed a master plan to transform Hanoi into a civilized and modern metropolis by 2030 (MoIT, 2012; MoPI, 2011). An important aspect of the urban planning regards the local food environment transformation, particularly food provision infrastructures, emphasizing retail modernization in which supermarket development is promoted and expected to increasingly replace more traditional food vending structures such as open-air wet markets (Dries, Tyng, & Dao, 2013; MoIT, 2009; Moustier, 2006). This policy was instigated by serious public health concerns regarding the agrochemical and bacteriological safety of the most commonly consumed fresh foods: fruits and vegetables, meats and fish products (Mai & Do, 2018). Supermarkets are regarded as powerful means to realizing improved food safety of fresh foods through private standards and traceability (Fuchs, Kalfagianni, & Havinga, 2011; Reardon, 2006; Wrigley & Lowe, 2010). To improve food safety and hygiene, policymakers in Hanoi stimulate retail modernization – or supermarketization – and since 2012, supermarket chains are rapidly developing in Hanoi.

More than 40% of the Hanoi population belong to lower-income groups living on less than 5 USD/cap/day (Nielsen, 2013). Despite lower income consumers aspiring to supermarket shopping (which is regarded attractive and offering better food safety guarantees), previous research in Hanoi rather demonstrated how these retail modernization policies lead to their exclusion (Wertheim-Heck, Vellema, & Spaargaren, 2014b; 2015). For the urban poor the progressing retail modernization is putting a healthy diet at stake. Supermarket development is dependent on private sector investment and as a consequence, supermarkets are concentrated in more affluent districts outside the action radius of most urban poor households. These modernization policies are designed to reduce and repress traditional fresh vending structures, including formal markets and informal fixed and mobile street vending, that poorer populations depend upon to access food (particularly fresh). Depriving the poor of their traditional access points for fresh foods may therefore, unintentionally, negatively impact their food choice and dietary intake. However, there have been no previous studies that evaluate the extent of these changes. A knowledge gap exists on how, and to what extent, transformations in the retail food environment have practical implications for food choice and dietary intake among the urban poor and its eventual impact on dietary quality and nutrition.

Food and nutrition insecurity is a well-recognized problem in Hanoi (Pulliat, 2013) and micronutrient deficiencies are particularly prevalent among women of reproductive age and young children (Laillou et al., 2012; 2014). Based on the 2015 definition of different food system typologies (Haddad et al., 2015), Vietnam can be described as ‘transitioning’, experiencing a significant improvement in living standards including a more diversified food supply and decreased rates of poverty and under-nutrition (Mishra & Ray, 2009; Thang & Popkin, 2004). Despite recent decreasing national trends in malnutrition, more than 3 million children under the age of five still suffer from malnutrition and 15% women diagnosed with chronic energy deficiency (NIN, 2012-2015). Yet at the same time, overnutrition is becoming more prevalent, with around 6 and 15% of children and adults overweight, and around 2% obese (MoH, 2016). There is little recent published data on the micronutrient intake and deficiency status of populations specific to urban areas, let alone specifically for Hanoi. As such, it is difficult to identify how the recent food environment changes have been reflected in diets and nutrition. The most recent nationally representative survey that captured micronutrient intake dates back to 2010, when retail modernization was just starting. It found that iron, zinc, vitamin A, folate and vitamin B12 were public health concerns, with children, women and the poor at higher risk for micronutrient deficiencies (Laillou et al., 2014, 2012; Nguyen, Nguyen et al., 2014). One of the primary causes of nutritional deficiencies is insufficient micronutrient intake from the diet, therefore food-based approaches that aim to increase the consumption of micronutrient rich foods are an appropriate strategy to improve micronutrient intake and decrease deficiencies (Nguyen Avula et al., 2014). In the context of food retail environment transformations, it is essential to measure the dietary intake quality of the urban poor in relation to the accessibility of micronutrient rich foods.

Continuing the rather one-dimensional, top-down, market driven policy focus on supermarket development could risk worsening the existing food and nutrition insecurity of the urban poor. Firstly, progressing supermarketization and the simultaneous repression of traditional vending structures, risks creating ‘food deserts’, or even better formulated ‘nutrition deserts’, for the urban poor as supermarkets present multiple access barriers (distance, time, physical accessibility, diversity of fresh foods available). Research in western societies has documented the potential harmful consequences of fresh food deserts on dietary intake, particularly in low-income neighborhoods (Hendrickson, Smith, & Eikenberry, 2006; Lewis et al., 2011; Markow, Booth, Savio, & Coveney, 2016; Pa'ez et al., 2010; Wang, Kim, Gonzalez, MacLeod, & Winkleby, 2007) and has indicated how fresh food retail interventions might result in dietary improvement (Wrigley, Warm, Margetts, & Whelan, 2002). These studies highlight the effects of different modes of retail food provision on diets. Secondly, the impact of supermarketization on dietary intake of the urban poor in particular, is still unclear. Although, supermarkets are assumed to offer better food safety and hygiene standards for fresh produce (Kantamaturapoj, Oosterveer, & Spaargaren, 2013), the broad assortment of processed foods offered in supermarkets is demonstrated to negatively impact the intake of nutritious fresh foods with a subsequent negative impact on nutrition and health in developed and lesser developed countries (Canella et al., 2014; Hyseni et al., 2017; Luiten, Steenhuis, Eyles, Mhurchu, & Waterlander, 2015; Moore, Diez Roux, Nettleton, & Jacobs, 2008; Poti, Braga, & Qin, 2017, 2015; Rauber, Campagnolo, Hoffman, & Vitolo, 2015; Taillie, Ng, & Popkin, 2015). In Thailand supermarket shopping was associated with increasing consumption of six ‘problem foods’ (soft drinks, snack foods, processed meats, bakery items, instant foods and deep-fried foods), while frequent fresh market shopping was associated with increased vegetable intake (Banwell et al., 2012; Kelly, Seubsman, Banwell, Dixon, & Sleigh, 2014). This is a problem, since dietary risk factors including low vegetable and fruit intake and the increased intake of fats, sugar, salt and ultra-processed foods are among the leading risk factors for NCDs (Gakidou et al., 2017). Thus, with a focus on the urban poor, specifically women, our study was designed to addresses two interrelated responsibilities of policymakers: (1) to realize socially all-inclusive food retail infrastructures and policies, and (2) to ensure that these infrastructures contribute to a safe and healthy diet for the population as a whole.

### 1.2 Addressing the knowledge gap

Multiple studies address the relationships between dietary intake and disparities in local food environments (Alkon et al., 2013; Black, Moon, & Baird, 2014a; Chen & Clark, 2013; Lamichhane et al., 2013; Leite et al., 2018; Murphy, Koohsari, Badland, & Giles-Corti, 2017; Samanta, 2015; Widener, 2014), in which most define the healthiness of the diet and the food environment in terms of nutritional quality and diversity. These studies leave the urgent issue of food safety in the emerging economies of the global South largely undiscussed (Mai & Do, 2018; Ortega & Tschirley, 2017). With the ultimate goal of contributing to realizing equal access to both safe and nutritious foods, the question is, can equitable access to safe and healthy foods be harnessed on the current normative retail modernization pathway?

This paper comprehensively describes our research approach that aims to rapidly provide insights to enable policy makers to design more effective inclusion food safety policies that don't have unintended consequences on other aspects of diet quality. The approach is novel in that it combines nutritional and food safety aspects in the notion of dietary quality in respect to food environment, but also in its theoretical and methodological approach. Firstly, it is innovative in its theoretical mix of combining dietary and social practices research; connecting dietary quality measurements to patterned behavior beyond rational choice. Secondly, in its holistic mixed method approach, which not only combines quantitative and qualitative methods, but also includes participatory methods to directly give voice to the urban poor. The objective of the mixed-method approach is to deconstruct the complexity and tension between two aspects of diet quality - food safety and nutrient content - to rapidly provide useful information to policy makers to move towards more inclusive urban food environments.

In the next section we describe the theoretical foundation and methodological approach. Then we proceed with highlighting some of the main research findings followed by a reflection on our approach.

## 2 Theory - A social practices approach to understand dietary intake

This research serves to enlarge the capacity of local authorities to plan and implement all-inclusive food–safe and nutrition–sensitive food retailing infrastructures. It links diet outcomes - in terms of nutritional quality, diversity and food safety-, with the food retail environment through food purchasing practices. Therein we acknowledge that food-purchasing practices are influenced by shifts in the system of food retail provision, urbanizing lifestyles, performance capabilities and different meanings of food.

Retail modernization policy is effectuated as a food safety motivated behavior change strategy. The central notion is that even deeply ingrained habits and routines can be altered through the reasoned adoption of alternatives. However, in emphasizing rational choice and reflexive, conscious and deliberate action, approaches like the normative supermarketization strategy, underestimate the importance of wider contextuality (Hindess, 2016). Everyday food consumption is far from simply being an expression of individual choice. Social Practice Theory offers an alternative that shifts the unit of analysis away from the individual to recognizable practices, like the routine of shopping for food, providing more grounded, situational perspectives on behavior change processes (Reckwitz, 2002; Shove, 2010; Spaargaren, , Lamers, , & Weenink, 2016; Strengers, Moloney, Maller, & Horne, 2015; Warde, 2016).

Social practices, like routinized, and habitual shopping for food, are structured entities composed of materials, meanings and competences, which are performed repeatedly within a social context (Shove, Pantzar, & Watson, 2012). In conducting research, it is vital to distinguish between practices as entities and practices as performance. Practices can only be empirically studied in the form of practice as performance (Welch & Warde, 2015). The performance enacted in specific moments and places, functions as a reading glass to obtain insights on practices as an entity. Practices as entities, such as shopping for food, persist even when the actual performance, the way of doing, evolves over time (Wertheim-Heck & Spaargaren, 2015). Moreover, it is important to highlight that practices are not performed in isolation. Rather, practices are bundled with other practices, for instance the interlinked practices of food shopping and commuting from work to home: ‘Practices link, inter alia, through shared ends or chains of action’ (Schatzki, 2016, p. 5). Thus, a change in the one practice, e.g. different commuting pattern to changed job location, often changes the other bundled practice, e.g. shopping for food.

Although practices theory-based research is expected to deliver valuable insights into the actual performance of daily life, its practical contribution to policy formulation, like in the case under study on retail modernization, is the subject of much debate. The biggest issue concerning the development of practice theory-based learning is its inherent complexity, and by not providing clear directions based on measured outcomes - the dominant foundation for evidence-based policy - which is argued to make it impractical. Since our study aims to inform policy makers, it was designed to combine more conventional quantitative approaches to food environment and dietary health and nutrition studies, with social practices approaches to food consumption; expanding the evidence-base beyond measuring and mapping to provide for in-depth understandings and capture shifts over time.

### 2.1 Conceptual framework

**Fig. 1:**
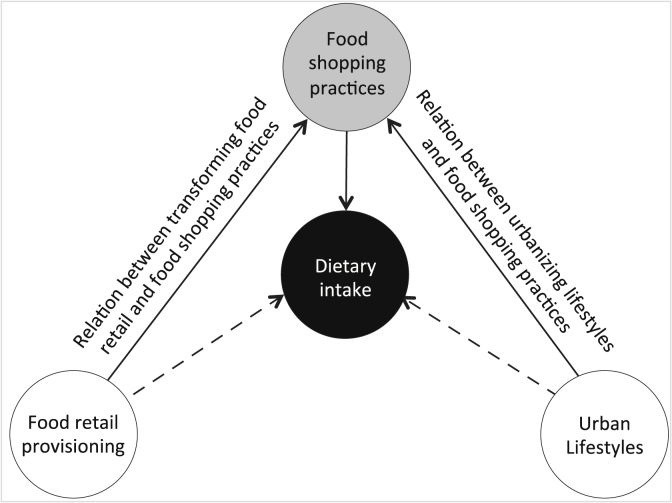
Conceptual framework.

We approach household food shopping and consumption, and thus subsequently dietary intake, as embedded in the organization of everyday life and within the immediate food retail environment. Thus, moving beyond conventional ‘rational choice’ approaches, which view food consumption primarily as the result of conscious and deliberate choice making by individual buyers. In this study we combine dietary research with a social practice-oriented perspective by including the habitual nature of food purchase and consumption (Shove, 2010; Warde & Southerton, 2012) and assessing how this is affected in a transforming food retail environment. Building on these social practices approaches to consumption, our research does not limit itself to a direct causal relation between diets and food retail, but incorporates the logics of everyday life to conform our conceptual framework below (Fig. 1). This study reasons from a household practices perspective, which considers the public and private stakeholders that constitute the system of provision (infrastructural and regulatory framework of household consumption practices and subsequent dietary intake) as contextual environment. Central in the design is dietary intake, which is assessed as the dependent variable of primarily food shopping practices and secondary food retail provisioning and urban lifestyles. Food shopping practices are considered an independent variable in relation to dietary intake, but are assessed as dependent variable in relation to food retail provisioning and lifestyles. Food retail provisioning and lifestyles are both considered independent variables, within this framework.

Along our conceptual framework we developed a hypothesis driven research set-up (see Table 1). The main underlying hypothesis is that diversified retail provision (acknowledging the value of traditional fresh food vending structures) will contribute to more diversified and nutritionally balanced diets; in other words, dietary diversity requires retail diversity, particularly for the urban poor. In this respect dietary intake is the dependent variable of food retail provisioning. In this research, however, we go a step further, by linking the food retail infrastructures with the actual practices of accessing food on the basis of urbanizing lifestyles, by means of multivariate research hypotheses testing.

**Table 1:** Multivariate research hypotheses overview.

Variables	Issue	Hypothesis	Main hypothesis
Dietary intake	Poor diet quality and high prevalence of micronutrient deficiencies among the urban poor, particularly women of reproductive age and young children.	Nutrition insecurity is related to access to dietary diversity and food safety, and nutritional knowledge and attitudes.	Diversified retail provision (acknowledging the value of traditional fresh food vending structures) will contribute to more diversified and nutritionally balanced diets; in other words, dietary diversity requires retail diversity
System of provision (SoP)	Food retail transformations are demonstrated to socially exclude urban poor populations; there exists a service delivery failure.	Access to dietary diversity and food safety is related to SoP (access to and type of retail food provisioning)	
Food shopping practices	Income is an important factor influencing consumer access to food, but time/spatial characteristics, household composition, social/family networks and cultural characteristics as well.	Practical access to retail food provisioning for consumers is related to the organization of everyday life.	
Lifestyles	Urbanization and modernization are leading to fundamental lifestyle changes, which include shifts in household composition and occupation, and subsequent shifts in cultural meanings and understandings.	The organization of everyday life including food purchasing and food preparation is importantly shaped by lifestyle changes	
Food safety risk perception and trust	Food safety is a prominent concern among all urban consumers, but formalized and officially guaranteed food safety systems are focusing on modern retail infrastructures	Formalized food safety guarantees are out-off reach of the urban poor	
Nutrition knowledge and attitudes	There is limited nutritional knowledge and positive attitudes towards the importance of selecting healthy and nutritious foods	Correct knowledge and positive attitudes are related to positive dietary quality and practices.	

Within our research we specifically address food safety and nutrition within the context of diets. Understanding food safety risk perceptions and trust in food safety as well as nutrition knowledge and attitudes are a useful for gaining insight into peoples' personal determinants of their food shopping and dietary habits. Food safety risk perceptions are demonstrated to impact the trust in food safety and ultimately the shopping practices in what products are when being purchased from whom or where (Wertheim-Heck, Spaargaren, & Vellema, 2014a). Further, knowledge has long been utilized as the foundation that can contribute to shaping an individual's attitudes, and ultimately behavior in health promotion (Bettinghaus, 1986). As such, knowledge about healthy, diverse and safe foods – and the retail food outlets from which these can be sourced - are necessary to encourage healthy diet consumption (Spronk, Kullen, Burdon, & O’Connor, 2014). However, knowledge alone is often insufficient to influence dietary behavior, as individual's attitudes influence future behavior no matter the individual's knowledge and help explain why an individual adopts one practice over alternatives, particularly when the correct knowledge is held (Kigaru, Loechl, Moleah, Macharia-Mutie, & Ndungu, 2015; Shepherd & Towler, 1992). Both nutrition knowledge and attitudes are considered independent variables that relates to food purchasing practices and dietary quality.

### 2.2 Scope

Two lower income urban districts in Hanoi were purposely selected based on the relative high percentage of low-income groups; excluding the most recently developed suburban areas to avoid a rural bias. Within these districts, the research specifically focused on women, since women are primarily responsible for household food purchasing, preparation and allocation, and are often the most nutritionally-vulnerable within the household. Nutrient deficiencies are particularly prevalent among women of reproductive age. Being responsible for caring for their families, women often prioritize food quality for others over themselves: working husband, sick and elderly in the household and their children (Wertheim-Heck, Anh, My, Klaver, & Huong, 2014c). This can result in inadequate diets, which can have serious impacts on women's health and wellbeing, and especially for the development of children during pregnancy and breastfeeding. Women are thus key-actors in understanding and addressing nutrition vulnerability.

In assessing dietary quality, all individual foods and drinks consumed were included, however in assessing the healthiness of the food retail environment more in-depth data were only collected on the specific fresh food category of vegetables. The reason for this in-depth focus on vegetables is that vegetables are critical for good health and nutrition and are an integral part of the traditional Vietnamese diet. They are affordable and easily accessible, yet on average, Vietnamese people do not consume sufficient quantities to meet WHO recommendations (Bui et al., 2016). Unfortunately, these and other fresh foods are amongst those of most concern by the government and consumers regarding food safety (Mai & Do, 2018).

## 3 Methods – a sequential mixed-method design

**Fig. 2:**
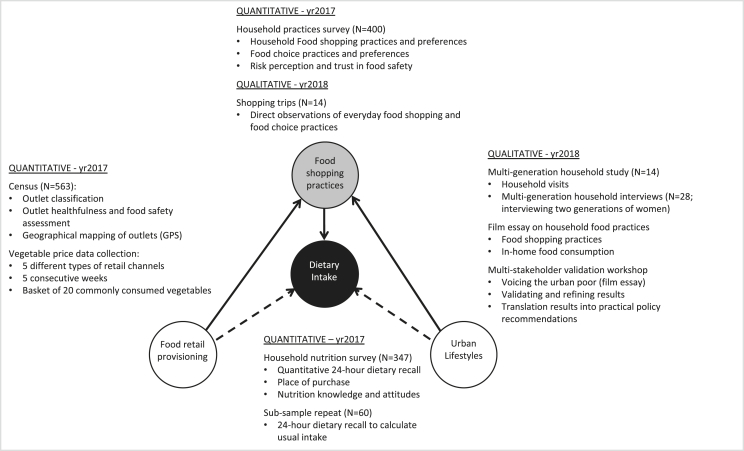
Methodological framework.

In this research we deployed a balanced sequential quantitative-qualitative research design. During the first year of our research we deployed quantitative methods to measure dietary outcomes in relation to the food retail environment and food shopping practices. The second year we used qualitative methods to obtain more refined insights on daily food routines and how these are co-constituted by the changing food environment. Seeking to explain our measured results and deepen our understandings, we paid specific attention to lifestyle and household dynamics, the respondents’ own understandings of nutrition and food safety, and the trade-offs they make in balancing food shopping and preparation with other activities in their everyday lives. Below we describe in detail the various methods deployed and Fig. 2 provides an overview of how our methods are linked to our conceptual framework.

### 3.1 Quantitative research

In seeking to understand how access to food retail outlets impacts dietary intake, four consecutive quantitative methodologies were used to collect data: Food retail outlet census, price data collection, household practices survey, and household nutrition survey (Fig. 2). IRB approval for the study was received from Hanoi Medical University, Vietnam in June 2017 (IRB00003121).

**Fig. 3:**
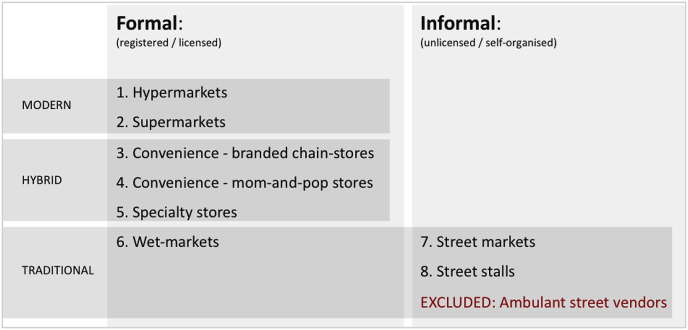
Retail census categorization.

*Census*. The study started out with a retail census - a street-by-street mapping of commercial food outlets - to obtain a detailed overview of the food provisioning structures. Each outlet was categorized along predefined retail typologies (Fig. 3), making a distinction between formal - licensed food retail business - and informal - self-organized unlicensed food retail business -, and between modern, hybrid, and traditional outlets. Within the formal food retail system, we further distinguished between four different types of retailing: (i) hyper-and supermarket retail, (ii) convenience retail, (iii) specialty retail focusing on a specific food category such as green grocers, bakeries and butcher's shop, and (iv) wet-market retail.

For each outlet the health dimension of the food assortment was assessed based on the level of processing (Monteiro et al., 2016) and nutritional value of all foods offered with a focus on vitamin A (FAO & FHI 360, 2016), and the food safety guarantees and claims for a range of specific minimally and un-processed vegetables. All retail outlets identified - including their detailed characteristics - were recorded in a database and were geographically mapped using Google visualizer (the interactive map is accessible here).

In addition, during 5 consecutive weeks, price data were collected of a preselected basket of twenty commonly consumed vegetables across different types of retail channels within the selected districts, which allowed for cross channel price indexing and making correlations with household shopping practices, income and food budget, captured in the survey described below.

**Fig. 4:**
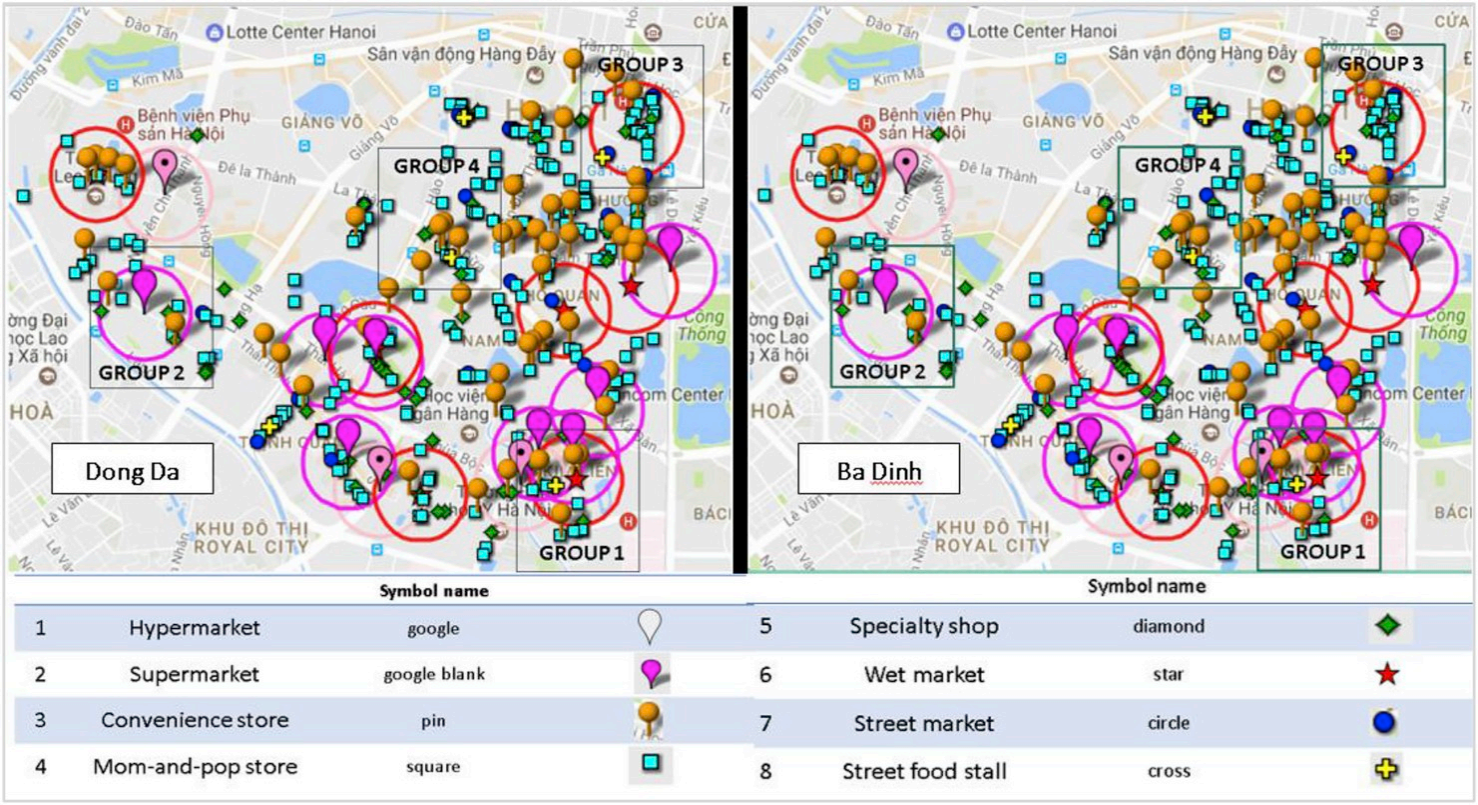
–Area based sampling on strata groupings in each district.

*Sampling*. Next, we moved to the quantitative household surveys and 24-h dietary recall. To understand the dietary impact of the food retail environment on the urban poor, we deployed a participant stratification strategy based on supermarket and wet market availability within the respondents’ 300 m walking distance action radius from the home (Table 2). This stratification was rationalized as these two retail outlets are the main focus of the Vietnamese retail modernization policy (MoIT, 2009). The results of the census mapping were used to group geographic areas within the two districts to meet the criterion of the four strata described in Table 1 and illustrated in Fig. 3 below. We developed sampling maps with clear action radius around known wet markets and supermarkets in the selected districts (Fig. 4).

**Table 2:** Stratification based on retail outlets within walking distance (300 m)^2^ from the home.

Respondents	Supermarket within walking distance	Formal wet-market within walking distance
Group 1 (N = 100)	Yes	Yes
Group 2 (N = 100)	Yes	No
Group 3 (N = 100)	No	Yes
Group 4 (N = 100)	No	No

A door-to-door sampling strategy was deployed for randomized inclusion of households with the following criteria: gender (women only), age (child bearing age, born after 1966), residency (at least 2 years at the address), household size (excluding single-person households), income (per capita daily income below 5 USD^1^) and role in household food acquisition (primarily responsible), the full sampling method is available as SI-1. Full informed verbal consent procedures were followed. After initial sampling and conduction of the Shopping Practices survey, households were verified that they did reside in the original allocated strata characteristics using GPS points collected. This resulted in a re-allocation of households, with the final strata sizes available in Table 3. Households were followed up for participation in the KAP Survey and 24-h dietary recall. There was an 87% response rate to the second follow up dietary intake survey (Table 3), with households not continuing mostly due to ill-health or re-location. A repeat 24-h dietary intake recall was conducted on a non-consecutive day on a subsample of N = 60 to allow for the calculation of usual intake.

**Table 3:** Respondent inclusion.

Strata No.	Targeted No of respondents	Actual inclusion	
		Household survey^a^	Combined nutrition survey and 24 h recall
1	100	84	71
2	100	95	81
3	100	92	84
4	100	129	111
Total	400	400	347^b^

a Inclusion redistribution over strata based on household GPS data and actual distance to nearest wet-market and supermarket.

b 87% response rate.

We based our sample size calculation on being able to detect a 10% difference in micronutrient intake (mg of iron (NIN, 2011)) of women across all strata. With a confidence of 95% power of 80% and a design effect of 2 (Gibson & Ferguson, 2008; Gorstein, Sullivan, & Begin, 2007), we concluded the sample size of 389 eligible HHs to be surveyed:

$$\;\text{n}=\frac{{\left(Z\alpha \;+\;Z\beta \right)}^{2}\times \left((P1\times \left(1-P1\right)\right)+\left(P2\times \left(1-P2\right)\right))}{{\left(P2\;-\;P1\right)}^{2}}\;DE=346$$

Zα - 95% confidence 1.645
Zβ - 95% confidence 1.645
P1 - baseline expected prevalence 12.3
P2 - endline expected prevalence 13.53
DE (design effect) default 2

The final sample size was increased to 400 to allow for even sampling across strata and to non-responsive households.

*Household Surveys.* Two quantitative household surveys were conducted. The first, household practices survey, assessed the food shopping/acquisition practices of the household, knowledge, attitudes and intentions towards food safety and the socio-demographic composition of the respondents. The second follow up nutrition survey was designed to assess the diet quality and the nutrition knowledge and attitudes of women within the household. The surveys served three main purposes: (1) discerning patterns in shopping practices to be interpreted against the census mapping; geographical mapping of practices with food shopping access points; (2) discerning patterns in shopping practices against diet quality; (3) discerning patterns in nutrition knowledge attitudes and intentions towards healthy and safe food acquisition and diet quality. For the development of both surveys the following sequence was followed: (i) Design: Besides the content, we developed questions and answers to make balanced choices on the use of Likert scales, forced choice and constant sum questions; (ii) Piloting: The questionnaires were individually piloted on a small sample of individuals to review the functionality and ability to capture relevant data as intended, paying careful attention to translation biases; (iii) Review: The questionnaires were reviewed and updated reflecting the outcomes in step (ii), piloting. The household practices survey was designed in six sections: screening according to the aforementioned inclusion criteria: gender, age, residency income., food shopping practices, and preferences, food item selection drivers, food safety and risk perception and trust in relation to vegetable shopping, and lastly a socio demographic section (full questionnaire is available as SI-2).

The nutrition survey contained two main components: the quantitative 24-h dietary recall and the nutrition knowledges and attitudes assessment. Dietary intakes of the women were assessed using an adapted version of the interactive 24-h recall method (Gibson & Ferguson, 2008) to also capture processing levels and the source of each ingredient consumed – focusing on which specific food retail outlet purchased foods are sourced from. All foods consumed were counted in the assessment, including out-of-home consumption (Lachat et al., 2009) and home-grown (Wertheim-Heck & Spaargaren, 2015). This allowed for a triangulation of ‘practice’ behavior with supermarket visits, regarding identifying which specific foods are sourced from where, to understand how potential changes in food retail outlets might impact on specific nutritious foods (or food groups). Currently there is very limited data available on the diets of the urban (particularly poor) population. As such, a quantitative 24 h recall was employed to go beyond simple qualitative measures of dietary diversity, to understand how the transforming food environment impacted the quality of the diet in regards to not only macro and micro nutrient intake but also the consumption of processed foods – given the important linkages to non-communicable diseases that is increasing in prevalence in urban areas in developing countries (Montiero, 2009). The 24hr recall also collected the species and varietal/breed level information to be able to calculate biodiversity in the diet and the source of each food or ingredient consumed (e.g. supermarket, wet-market, convenience store, own production etcetera). The nutrition knowledge and attitude questions were adapted from the Nutrition Knowledge, Attitudes and Practices (KAP) manual published by FAO (2014), and focused on knowledge and attitudes towards diet diversity, micronutrients, under and over nutrition. These questions identified underlying reasons as to why food choices occur.

*Analysis methods applied.* Five main diet quality indicators were used to measure the quality of the diet. Diet Diversity Score (DDS) was calculated as a count of the number of unique food groups out of 10 consumed, and Minimum Diet Diversity (MDD) which is a percentage of the population that consumed 5 or more food groups (FAO & FHI 360, 2016). Mean Nutrient Adequacy Ratio (MAR) was calculated as an arithmetic mean of a set of individual Nutrient Adequacy Ratios (NAR), calculated as quantity of a nutrient consumed per its requirement for each individual on a daily basis, capped at 1, to prevent compensation for those NARS with lower levels when calculating MAR (Lachat et al., 2018). Table 4 provides a summary of the main indicators utilized to measure nutrition knowledge and attitudes, diet quality and sources of food from the quantitative dietary intake data.

**Table 4:** Indicators used to analyse the nutrition survey.

Diet Quality	Description	Metric
Nutritional quality of the diet	Micro and macro nutrient quality of the diet	Mean daily intakes for a set of key nutrients
	Micro and macro nutrient quality of the diet	Usual mean intakes for a set of key nutrients
Women's Dietary Diversity Score	Food group diversity of the diet, micronutrient proxy	Count of the number of food groups consumed out of 10
Minimum Dietary Diversity Reached	Food group diversity of the diet, micronutrient proxy	5 or more food groups out of 10 consumed)
Dietary Species Richness	Biodiversity levels of the diet	Count of the number of unique species consumed
Ultra-processed food consumption	Relative contribution of ultra-processed foods in the diet	% Energy from Ultra processed foods
	Quantity of ultra-processed foods in the diet	Mean intake from ultra-processed foods (g)
Nutrition Knowledge and Attitudes:		Composite Indicator of the below, evenly weighted
	Micronutrient Knowledge	Count of correct answers
	Micronutrient Attitudes	Count of positive answers
	Diet diversity knowledge	Count of correct answers
	Diet diversity attitudes	Count of positive answers
	Under nutrition knowledge	Count of correct answers
	Under nutrition attitudes	Count of positive answers
	Over nutrition knowledge	Count of correct answers
	Overnutrition attitudes	Count of positive answers
Contribution of ultra-processed foods	Sources of ultra- processed foods per retail outlet typology	% of ultra- processed foods per retail outlet typology
	Quantity of ultra-processed food consumed	g of ultra- processed foods per retail outlet typology and % of total energy intake
Source of nutrients per retail outlet	Micro and macronutrients intakes, consumed from foods sourced from the different retail outlet typologies	% value of total daily nutrient intakes per retail outlet typology (
		Absolute value of nutrient intakes per retail outlet typology

Descriptive analysis was run for all data sets, comparing means between strata and from the population as a whole. In addition, forward step-wise linear models were used to explore relationships between nutrition knowledge and attitudes, dietary quality and the retail outlet source of foods consumed (see Table 4).^3^ Confounding variables were controlled for that are known to have an effect ton nutrition knowledge and diet quality including women's age, reproductive status, education level and income. The strata were analysed and compared to see if patterns or associations exists between dietary quality and variables related to access to different food retail outlets, and knowledge, attitudes and practices.

In addition, food list with consumption frequency per retail outlet were generated to develop an understanding of which types of consumed foods were sourced from where; which could also be used to triangulate information from the household food shopping practices survey.

### 3.2 Qualitative research

In the second year of our research we complemented our quantitative research with more in-depth oriented qualitative methods to include the wider contextuality of everyday food consumption and capture shifts over time. Our qualitative methods zoomed in on lifestyle and household dynamics in food coping strategies of households under precarious conditions within a transformative food retail environment. To capture shifts over time we focused on multi-generation households.

*Multi-generation household study.* The aim of the follow-up in-depth qualitative study was to understand how food security interacts with intra-household dynamics and non-food factors. Participants for the qualitative multi-generation research were selected from the sample surveyed in the quantitative research. This allowed for an in-depth and qualitative exploration of the underlying logics and influencing factors that could not be quantified; gaining insights on the reasoning and patterns of food consumption practices within the organization of everyday life and specifically also capturing changes over time and across generations. Criteria for households selection were based on the inclusion criteria that the respondent had indicated in the first survey to i) live in a household with a woman from an older generation and ii) willing to participate in the more in-depth multi-generation research (N = 77). Out-off this sample 35 households were selected based on variation in socio demographics (age and household composition) and in food shopping practices (frequency, moment and retail channel), and in distribution across strata and. Our aim was to include 4–5 households per strata but in the end 14 households that could be reached were included (14 households could not be reached, and 7 households refused to continue due to time-constraints); 5 households from stratum 1, 4 households from stratum 2, 3 households from stratum 3 and 2 households from stratum 4. Since our quantitative research revealed similar shopping practices across data (see findings below) the uneven distribution across strata was not considered a constraint in our more in-depth qualitative investigation. The final sample reflected the aimed for socio-demographic variation.

The interviews were conducted at the respondents' homes. This allowed for assessing the cooking and eating space(s) within the home and to capture information on the home environment relevant for the food consumption practices. We interviewed the main respondent and her mother (-in-law) in a joined interview to capture also contrasting feelings, perceptions and opinions related to food consumption practices within the household over time. Although allowing for interactions, we started the interview with the main respondent and followed with the mother (in-law). We didn't encounter biases by respondents providing socially desirable responses, which is explained by the fact that food is a popular topic for conversation in Vietnam and the women interviewed were appreciative to be consulted on their everyday food experiences. In addition, interviews took place in a natural setting for a duration of 1,5 h on average, with ample opportunity for cross checking. Moreover, the shopping practices visits allowed for triangulation of ‘doings’ and ‘sayings’. The interviews were semi-structured to allow for free-flow but also ensuring all relevant topics were addressed. The interviews explored the gender and generation relations and responsibilities within the household and how these have shifted over the past 5–10 years; how urbanization and modernization result in changes in family forms and gender roles in relation to food consumption practices. ‘How women are balancing nutrition and food safety; Experiences with food (in)security at the household – resource constraints and in what way the household prioritizes food over the acquisition of other products and services -, and the individual level - intra-household food allocation. In interviewing the older generation, we deployed a life history/life cycle approach to obtain a detailed understanding of the trajectory upon which food consumption practices have travelled to reach its present routines; capturing lifestyle events and changes in the food environment to uncover food consumption practice trajectories across time and their sustaining elements despite ‘crisis-like’ conditions. In addition to the home-based interviews the main-respondent was joined during a routine shopping trip; delivering observational insights on the actions and decision ‘from home – back to home’ to contextualize and provide further depth to understanding the everyday practices.

*Film essay.* With the aim to inform policymakers and enlarge their capacity in designing all-inclusive food safety strategies, we also portrayed the everyday in-home and shopping practices in a film-essay, first hand voicing the urban poor who are not easily consulted in policy design. We selected three women to be included in the documentary based on their consent to be filmed and their variation in household composition and setting and food consumption practices. We approached the making of the film-essay by using the filming as video-based fieldwork (Goldman et al., 2009). Collecting ‘naturally’ occurring data focusing on the structures of interaction and social and behavioral mechanisms in food consumption activities. The filming allowed us to record the ongoing interaction of people in a specific context and all aspects of the environment that structure these interactions, while preserving the temporal and sequential structure, which is characteristic of interaction (Knoblauch, , Schnettlet, , Raab, , & and Soeffner, 2006). The final script was written when the footage was shot based on what we uncovered during the research (and with the camera). However, we developed an outline at the beginning as a guide to point the camera team in the right direction (Derry et al., 2010). The film essay titled ‘Retail diversity for dietary diversity - food safety and nutrition for the urban poor’ is accessible here.

*Multi-stakeholder validation workshop*. Lastly, the results from the research were shared during a multi-stakeholder workshop which through a ‘constructive confrontation design’ stimulated participants to actively reflect and complement on the research. During the workshop the film-essay was used to voice the urban poor that felt too shy or simply did not have the time to participate. The documentary enhanced collaborative commentary, critique and conversational exchange for possible re-interpretation of the research results. Including the views of multiple stakeholders, strengthened the policy brief which contains an agenda for careful considerations in advancing the retail modernization process and future orientation for ensure all-inclusive food systems.

## 4 Findings

Below we present limited, though the major, findings of our research to allow for reflection on the methodology. More detailed reporting on the study results is (and will be) reported elsewhere (Raneri & Wertheim-Heck, 2019; Wertheim-Heck, Raneri, & Oosterveer, 2019).

The census captured the existing diversity in food retailing which confirmed the uneven geographical distribution of the various retail outlets. Wet-markets are geographically more evenly distributed across the districts, compared to hyper- and supermarkets. Secondly, supermarkets offered a similar assortment of fresh fruits and vegetables as (in)formal markets, but with visual food safety claims and certificates, which were not observed in the open-air based markets. However, supermarkets also offered a wider selection of less healthy ultra-processed foods. Thirdly convenience retail channels were most widely distributed, accounting for 67% of all food outlets. The census exposed an important distinction between the more modern chain-stores and traditional mom-and-pop stores: 62% of the chain-stores offered fresh vegetables, versus only 2% of the mom-and-pop stores.

Although it was expected that dietary quality would be influenced by geographical proximity to different formal retail outlets, our analysis of the 24-h dietary recall showed no significant difference in dietary quality across the different strata. Overall, diet quality was minimal. On average MAR was only 0.54, meaning that women consumed just over half of their daily nutrient requirements. MDD was reached by 75% of women (FAO & FHI 360, 2016) with an average DDS of 5.3 food groups consumed (only 0.3 of a food group above the inclusion cut off of 5 food groups). The quantitative knowledge and attitudes survey found consumers were aware that nutrition is important and have basic knowledge and understanding of nutrition concepts. The in-depth interviews with the women confirmed this, as almost all respondents expressed the importance to have good nutrition and diets and the importance of fresh and safe vegetables to do so. Foods purchased from traditional retail outlets helped maintain healthy diets and contributed most to daily nutrient intakes: 70% protein; 56% energy; ± 80% vitamin A and C and ±70% calcium, iron and zinc. Foods sources from modern retail outlets made up a very small percent of the diet and did not contribute significantly to the quality of the diet. A limited amount of foods (19%) were purchased in supermarkets and convenience outlets, however, these outlets contributed to 84% of the ultra-processed foods consumed.

Nearly all (90%) of households still preferred to shop at traditional (in)formal markets (wet-markets and street-markets), triangulating the data form the diet recall which found 70% of foods were sourced from these outlets. The study revealed that supermarkets and convenience stores offer a higher percentage and wider range of ultra-processed than traditional open-air markets and that they were frequented mainly for purchasing and consuming these less healthy foods. The urban poor did not utilise supermarkets or convenience stores for primary, daily fresh food, grocery shopping even when these modern outlets were located close to home. When a wet-market was beyond walking distance, consumers chose informal street markets rather than modern retail outlets. Unfortunately, street vending is unregulated and more unhygienic than formalized wet markets, meaning potentially increased food safety vulnerability.

The main drivers of continued shopping in traditional (in)formal markets, driven by preferred shopping practices including the diversity and perceived freshness of products offered, convenient location, overall enjoyment of open-air market shopping, availability of healthy foods, lower food-price and perception on trusted food-safety. Social considerations were also raised including habitual nature and worrying about maintaining the culture and tradition associated with market shopping.

### 4.1 Testing the theoretical framework linking – KAP – diet – food sources

The results of the linear models ran to test these models are reported in detail elsewhere (Raneri et al., under development). Nutrition knowledge and attitudes influenced only a few aspects of diet quality (Table 5) particularly for Dietary Species Richness and intakes on key food groups (vitamin A rich fruits and vegetables, legumes, starchy staples) and micronutrients (folate, magnesium and sodium). However, these effects were small and not likely to be significant when contextualized to making recommendations for improving nutrition knowledge and attitudes to achieved better diet quality outcomes.

**Table 5:** Linear relationships between nutrition knowledge and attitudes and dietary quality.

	Nutrition Knowledge^a^			Nutrition Attitudes		
	Coef.	Std. Err.	P > t	Coef.	Std. Err.	P > t
Dietary Species Richness^a^^1^	0.5007918	0.1936358	0.01	0.630657	0.30531	0.04
vitamin A rich fruits and vegetables1				25.33005	11.14633	0.024
Legumes^a^	21.55172	7.251786	0.003			
Starchy staples**	−67.51536	29.78622	0.024			
Folate^a^	36.92938	13.25238	0.006			
Magnesium^a^	24.19762	11.69268	0.039			
Sodium	−368.7091	111.8961	0.001			

a Micronutrient Knowledge for all indicators except for Starchy staples, for which significant results were found for Overnutrition knowledge.

The multi-generation interviews uncovered that the urban poor don't feel empowered in voicing their needs and concerns in coping with food safety and nutrition in everyday life. Consumers understood the government's rationalization that the food retail needs to modernize. However, they still wanted to keep wet-markets as part of their daily food environment and expressed concerns about the current rather one-dimensional direction, and their ideas about more hybrid alternatives that involved the co-sharing of responsibilities in managing food safety at the markets. The interviews also provided insights that explained why supermarkets are not utilized more frequently – specifically regarding lack of trust in the safety claims. These were based on own experience, ranging from having worked at a supermarket and experiencing first-hand relabeling fresh produce, or having seen how a regular market vendor is also delivering fresh foods to the supermarket. A pattern in which the elderly women interviewed appeared more positive about the future potential of supermarkets than their daughters (in-law) was brought to light. For the latter the supermarkets are too inconvenient and do not fit the urban lifestyle of working women in multiple ways. Similar to what was found in the household food shopping survey, women identified that time was a constraint in regard to opening hours and time required to go through a supermarket: parking, weighing, and waiting at the check-out counter. In contrast, markets are open early morning and vendors offer all kinds of convenience services, like cutting and cleaning.

The interviews uncovered shifts in food consumption practices that were not captured in the 24 h diet recall. All households mentioned the importance of having multiple dishes at the dinner table, catering to the diversity in tastes and preferences of the household members. This not only included the adults in the household, but most importantly the children. All women interviewed indicated to strongly align their food choices with the tastes and preferences of their children because children would eat better when they like the food. However, in the context of rapid changes in the urban food environment beyond retail, including out-of-home and westernization of the local food system, food preferences of the younger generation are shifting away from being traditional healthy, fresh and plant based to increasingly include fast-food style dishes like pizza and fried chicken. The urban poor can't afford eating out but are increasing incorporating home-made fast-food in the household diet, or to buy the ready-made alternatives available in supermarkets. For the time being, the ingredients to prepare these foods are still being purchased from traditional markets, which is likely why the transition was not captured in the 24 h diet recall when analyzing the source of foods consumed. Despite most women achieving high scores in both knowledge and attitudes towards the seriousness of overnutrition in the quantitative study, and again in the interviews women appeared to be aware of the food health risks related to fried dishes and soft drinks, food choice is one of the few luxuries they can allow their children. Catering to their children's tastes, several women mentioned serious overweight among their children.

## 5 Discussion

Our research approach sought to deliver insights on how the current retail modernization pathway of the urban food environment is impacting the dietary intake and hence quality of the urban poor. We argue that in order to provide clarity on the trade-offs between improving food safety through retail modernization strategies and ensuring equitable access to, and consumption of, safe and nutritious foods requires integrative thinking; linking qualitative data of people's shifting lifestyle practices and preferences to quantitative data on the situation as it is. In this study we combined quantitative nutrition science with social practices theory informed approaches.

Benefits of the mixed-method approach include being able to rapidly capture aspects of the food environment - especially the transitions -, through the complementary information collected in the quantitative and qualitative methods applied. Specifically, the ability to triangulate food shopping practices, that wouldn't have been captured with the cross-sectional quantitative methods alone. The dietary intake data collected with the quantitative 24 h recall was not able to extrapolate that diets were being driven to change. The qualitative information provided the insight that whilst women's diet quality had not yet changed, children's diets were changing and actually driving shifts in how food was being prepared at home. Without the use of the mixed methods, incomplete and potentially inaccurate information would have been generated. Whilst this is of course the nature of cross-sectional data, it was expected that the proximity of the retail outlet would allow for cross-comparison between different aspects of the population.

Ideally, a longitudinal study design would be applied using quantitative data methods to rigorously assess diet quality changes over time. However, this is expensive and exceptionally time consuming. The use of a mixed-method approach offers an alternative to costly longitudinal studies, in response to rapidly generating relevant information and evidence to inform policy makers on urgent issues that require immediate action and cannot afford the luxury of waiting for repeat longitudinal studies to be completed.

The application of a mixed-method approach that not only integrates qualitative and quantitative methods but also transcended disciplines provided a unique perspective that allowed for a novel tactic on how to address the complex research problem that similarly transcended multiple thematic scopes: food shopping practices, food safety, dietary intake and nutrition. The benefits of working in multidisciplinary teams are well documented, and in particular the ability of such teams to foster innovative and unique approaches and solutions are key (Mooney et al., 2013). Whilst challenges do exist, such as terminology, these are quickly overcome and can result in research outcomes and results that can be communicated simply and easily to non-technical audiences.

A limitation of the study design is the heavy response burden on participants, generated through the multiple follow up surveys required to generate sufficient data on both the shopping practices and nutrition dimensions of the study, as well as for the qualitative in-depth interviews. The repeat intake for the 24 h recall was postponed by nearly a year in an attempt to prevent overload, after the high non-response rate was observed for the first dietary recall following the shopping practices survey. Postponing the repeat intake was done as a risk-mitigation strategy towards jeopardizing the response for the qualitative aspect of the study. The trade-off is that data collection that enabled the calculation of usual intake is a year apart. However, considering that only households that reported a nil change in household income or diets and who had not participated in nutritional or health education programs were included, assisted with justification that these data could be used to calculate usual intake for that season, despite being nearly a year apart. Innovative 24 h recall methods that reduce respondent burden have been developed but are yet to be systematically validated or widely used (Illner et al., 2012).

Future research/applications of the methodology should consider increasing the heterogeneity in the sample; in this case the urban poor had similar shopping practices, nutrition knowledge and diet quality regardless of proximity to retail outlets, which did not allow for sub-population comparisons considering the proximity to retail outlets did not influence the outcome variables. Future research should include a wide scope of participants to compare, for examples populations from different socioeconomic brackets (low – middle and upper), as well as include additional variables known to drive of diet quality and consumer purchasing behavior.

### 5.1 Development relevance beyond Hanoi, Vietnam

Modernization of the food retail environment, with subsequently changes in dietary intake towards an increased consumption of processed foods, is a global phenomenon. Understanding nutrition in relation to changing retailing structures, with a specific focus on the most vulnerable group of urban poor women, informs interventions that improve the accessibility and eventually consumption of safe and healthy foods for the poor in general.

The applied method allows for the rapid generation of evidence, together with the buy in and validation from local authorities to build empathy and understanding on the impact of existing food safety policy on the urban poor. The capacity of local authorities to adequately plan and implement food retailing infrastructures and support services to cope with the challenges of urbanization and rapidly changing food needs of their populations has direct consequences for the food and nutrition security status of their citizens. Acknowledging the aforementioned challenges policymakers are facing, this research provides understanding on how the urban poor, within the organization of their daily lives, cope with progressing food retail environment transformations and how, and to what extent, these changes impact their daily dietary intake in terms of nutrition, diversity and food safety guarantees.

Currently, Vietnam has made significant progress in decreasing rates of undernutrition, however obesity and prevalence of NCDs is increasing – particularly in the urban areas – putting pressure on public health systems. While we found that the Urban poor are not yet utilizing supermarkets, the negative risk to diets and nutrition associated with changing the primary food environment to depend wholly on supermarkets is likely to either i) drive people towards more unhealthy diets and increased NCDs and/or ii) increase food insecurity due to increasing food budget expenditure to maintain the current diet quality. Although the often-unhygienic conditions and lack of adequate control mechanisms of traditional open-air markets are not contested, the limits of pushing modernization and banning informal retail structures without inclusive consultation of the urban poor is risking their food and nutrition security.

## 6 Conclusion

Getting back to our central hypothesis about retail diversity within the food environment: we measured the dependency of the urban poor on traditional and modern retail structures. Through traditional structures they are able to maintain minimum dietary adequacy. The cross-sectional dietary quality measurement did not uncover the process of dietary change. It was only through the qualitative research that we were able to uncover how in a changing food environment, children are importantly defining the household menu. This process can however be ascribed to changes in the wider food environment beyond retail modernization. The use of a mixed method approach that integrated qualitative and quantitative methods, and crossed multiple disciplines was able to rapidly deliver evidence. The use of a consultative local stakeholder meeting was effective in validating the results achieved with the method, as well as for identifying policy recommendations and actions to improve the existing food safety policy to resolve the social inequity issues, and to provide practical solutions for alleviating some of the tension on the food safety – nutrition burden faced daily by the urban poor.

## Footnotes

1 Socio-demographic triangulation for income underreporting: Income underreporting is a prevalent complication in poverty research and this does not exclude this research. Although we based the participant inclusion on a per capita income of <5 USD/day, the actual income level may be higher. To assess whether we included more vulnerable populations we benchmarked the average household size of 4.2 persons and 30% extended households in our research with the generic urban population statistics of Vietnam, which report substantial lower household sizes: Average household size 3.7 persons (GSO, 2009) and 14.9% extended households Guilmoto, C. Z., Loenzien, M. de (2015). "Emerging, transitory or residual? One-person households in Viet Nam." Demographic Research**32**: 1147–1176. This indicates that our household profile indeed corresponds with the profile of lower income classes.

2 With a focus on the poor, the action radius of 300 m from the home by foot was taken as marker for spatial accessibility. Previous research in Hanoi demonstrated how poorer populations depend on daily food budgeting due to daily fluctuating incomes and can't afford to spend on fuel and parking for shopping purposes and thus shop by foot (Wertheim-Heck et al., 2014c). This we benchmarked against how the radius is defined in international research: 800 m for supermarkets and 200 m for convenience stores, in which the latter refers to walking distance (Raja, Ma, & Yadav, 2008). Previous research included shopping trip by bikes (Wertheim-Heck 2014b). When bikes are used for shopping this remains within smaller distances from the home, within the ‘own neighborhood’ (important in this respect is the climate: many months are considered too warm for biking). Hanoi is a city with very limited public transportation options (Hansen, 2015) and public transportation is not used for shopping. Supermarkets have home-delivery services in Hanoi, but the delivery is not free and thus forms a potential affordability barrier for low income groups.

3 More detail information of the analyses will be provided in a follow-up methods paper.

## References

[bib1] Alkon A.H., Block D., Moore K., Gillis C., DiNuccio N., Chavez N. (2013). Foodways of the urban poor. Geoforum.

[bib2] Banwell C., Dixon J., Seubsman S.A., Pangsap S., Kelly M., Sleigh A. (2012). Evolving food retail environments in Thailand and implications for the health and nutrition transition. Public Health Nutrition.

[bib3] Bettinghaus E.P. (1986). Health promotion and the knowledge-attitude-behavior continuum. Preventive Medicine.

[bib4] Black C., Moon G., Baird J. (2014). Dietary inequalities: What is the evidence for the effect of the neighbourhood food environment?. Health & Place.

[bib5] Bui T.V. (2016). Fruit and vegetable consumption in Vietnam, and the use of a “standard serving” size to measure intake. British Journal of Nutrition.

[bib6] Canella D.S., Levy R.B., Martins A.P.B., Claro R.M., Moubarac J.C., Baraldi L.G. (2014). Ultra-processed food products and obesity in Brazilian households (2008-2009). PLoS One.

[bib7] Chen X., Clark J. (2013). Interactive three-dimensional geovisualization of space–time access to food. Applied Geography.

[bib8] Derry S.J. (2010). Conducting video research in the learning sciences: Guidance on selection, analysis, technology, and ethics. The Journal of the Learning Sciences.

[bib9] Dries M., Tyng G., Dao T.M. (2013). Retail foods. Sector report GAIN (global agricultural information network) Vietnam.

[bib10] FAO (2014). Guidelines for assessing nutrition-related knowledge, attitudes and practices.

[bib11] FAO, FHI 360 (2016). Minimum dietary diversity for women- a guide to measurement, food and nutrition technical assistance Ⅲ.

[bib12] Friel S., Baker P. (2009). Equity, food security and health equity in the Asia Pacific region. Asia Pacific Journal of Clinical Nutrition.

[bib13] Fuchs D., Kalfagianni A., Havinga T. (2011). Actors in private food governance: The legitimacy of retail standards and multistakeholder initiatives with civil society participation. Agriculture and Human Values.

[bib14] Gakidou E., GBD 2016 risk factors collaborators (2017). Global, regional, and national comparative risk assessment of 84 behavioural, environmental and occupational, and metabolic risks or clusters of risks, 1990-2016: A systematic analysis for the global burden of disease study 2016. The Lancet.

[bib15] Gibson R., Ferguson E. (2008). An interactive 24-hour recall for assessing the adequacy of iron and zinc intakes in developing countries. Harvestplus Technical Monograph.

[bib16] Goldman (2009). Video research in the learning sciences.

[bib17] Gorstein J., Sullivan P., Begin (2007). Indicators and methods for cross-sectional surveys of vitamin and mineral status of populations.

[bib18] Haddad L. (2015). Actions and accountability to advance nutrition and sustainable development. Globalizations.

[bib19] Hansen A. (2015). Transport in transition: Doi moi and the consumption of cars and motorbikes in Hanoi. Journal of Consumer Culture.

[bib21] Hendrickson D., Smith C., Eikenberry N. (2006). Fruit and vegetable access in four low-income food desert communities in Minnesota. Agriculture and Human Values.

[bib22] Herforth A., Ahmed S. (2015). The food environment, its effects on dietary consumption, and potential for measurement within agriculture-nutrition interventions. Food Security.

[bib23] Hindess B. (2016). Choice, rationality and social theory.

[bib24] Hyseni L., Bromley H., Kypridemos C., Flaherty M.O., Lloyd-williams F., Guzman M. (2017). Systematic review of dietary trans-fat reduction interventions. Bulletin of the World Health Organization.

[bib25] Illner A.-K., Freisling H., Boeing H., Huybrechts I., Crispim S.P., Slimani N. (2012). Review and evaluation of innovative technologies for measuring diet in nutritional epidemiology. International Journal of Epidemiology.

[bib26] Kantamaturapoj K., Oosterveer P., Spaargaren G. (2013). Providing sustainable food in urban Thailand. Journal of Sustainable Development Studies.

[bib27] Kelly M., Seubsman S., Banwell C., Dixon J., Sleigh A. (2014). Thailand's food retail transition: Supermarket and fresh market effects on diet quality and health. British Food Journal.

[bib28] Kigaru D.M.D., Loechl C., Moleah T., Macharia-Mutie C.W., Ndungu Z.W. (2015). Nutrition knowledge, attitude and practices among urban primary school children in nairobi city, Kenya: A KAP study. BMC Nutrition.

[bib29] Knoblauch H., Schnettlet B., Raab J., Soeffner H.-G. (2006). Video-analysis methodology and methods: Qualitative audio-visual data analysis in sociology.

[bib30] Lachat C., Bao Khanh L.N., Cong Khanm N., Quang Dung N., Van Anh N.D., Roberfroid D. (2009). Eating out of home in Vietnamese adolescents: Socioeconomic factors and dietary associations. American Journal of Clinical Nutrition.

[bib98] Lachat (2018). Dietary species richness as a measure of food biodiversity and nutritional quality of diets. PNAS.

[bib31] Laillou A., Pham T.V., Tran N.T., Le H.T., Wieringa F., Rohner F. (2012). Micronutrient deficits are still public health issues among women and young children in Vietnam. PLoS One.

[bib32] Laillou A., Yakes E., Le T.H., Wieringa F.T., Le B.M., Moench-Pfanner R. (2014). Intra-individual double burden of overweight and micronutrient deficiencies among Vietnamese women. PLoS One.

[bib33] Lamichhane A.P., Warren J., Puett R., Porter D.E., Bottai M., Mayer-Davis E.J. (2013). Spatial patterning of supermarkets and fast food outlets with respect to neighborhood characteristics. Health & Place.

[bib35] Leite F.H.M., De Carvalho Cremm E., De Abreu D.S.C., Oliveira M.A.D., Budd N., Martins P.A. (2018). Association of neighbourhood food availability with the consumption of processed and ultra-processed food products by children in a city of Brazil: A multilevel analysis. Public Health Nutrition.

[bib36] Lewis L.B., Galloway-Gilliam L., Flynn G., Nomachi J., Keener L.C., Sloane D.C. (2011). Transforming the urban food desert from the grassroots up: A model for community change. Family & Community Health.

[bib37] Luiten C.M., Steenhuis I.H., Eyles H., Mhurchu C.N., Waterlander W.E. (2015). Ultra-processed foods have the worst nutrient profile, yet they are the most available packaged products in a sample of New Zealand supermarkets. Public Health Nutrition.

[bib38] Mai H.T., Do K.H.P. (2018). Consumer concern about food safety in Hanoi, Vietnam. Food Control.

[bib39] Markow K., Booth S., Savio S., Coveney J. (2016). Improving access to community-based food systems: Comparing perspectives of low socioeconomic individuals and food system representatives. Nutrition and Dietetics.

[bib40] Mathur O.P. (2013). Urban poverty in ASIA.

[bib41] Mishra V., Ray R. (2009). Dietary diversity, food security and undernourishment: The Vietnamese evidence. Asian Economic Journal.

[bib42] MoH (2016). National survey on the risk factors of non-communicable diseases (STEPS) Viet Nam, 2015. http://origin.who.int/ncds/surveillance/steps/VietNam_2015_STEPS_Report.pdf.

[bib43] Mohiddin L., Phelps L., Walters T. (2012). Urban malnutrition: A review of food security and nutrition among the urban poor. Nutrition Works.

[bib44] MoIT (2009). Overview of the current status and direction of economic and social development in Hanoi in 2010, with a vision to 2030.

[bib45] MoIT (2012). No. 1758/QD-TTg 20 nov 2012.

[bib46] Montiero (2009). Nutrition and health. The issue is not food, nor nutrients, so much as processing. Public Health Nutrition.

[bib99] Monteiro C., Cannon G., Levy R., Moubarac J.C., Jaime P., Martina A.P., Canella D., Lousasa M., Parra D. (2016). NOVA: the star shines bright. World Nutrition.

[bib47] Mooney S., Young D., Cobourn K., Islam S. (2013). Multidisciplinary research: Implications for agricultural and applied economists. Agricultural & Applied Economics.

[bib48] Moore R. (2013).

[bib49] Moore L.V., Diez Roux A.V., Nettleton J.A., Jacobs D.R. (2008). Associations of the local food environment with diet quality--a comparison of assessments based on surveys and geographic information systems: The multi-ethnic study of atherosclerosis. American Journal of Epidemiology.

[bib50] MoPI (2011). Hanoi's master plan officially publicized. http://www.mpi.gov.vn/en/Pages/tinbai.aspx?idTin=3136.

[bib51] Moustier P. (2006). Trends and policy on markets and supermarkets in Vietnam.

[bib52] Murphy M., Koohsari M.J., Badland H., Giles-Corti B. (2017). Supermarket access, transport mode and BMI: The potential for urban design and planning policy across socio-economic areas. Public Health Nutrition.

[bib53] Nguyen P.H., Avula R., Ruel M.T., Saha K.K., Ali D., Tran L.M. (2014). Maternal and child dietary diversity are associated in Bangladesh, Vietnam, and Ethiopia. Journal of Nutrition.

[bib54] Nguyen P.H., Nguyen H., Gonzalez-Casanova I., Copeland E., Strizich G., Lowe A. (2014). Micronutrient intakes among women of reproductive age in Vietnam. PLoS One.

[bib55] Nielsen (2013). Vietnam pocket reference book. http://www.nielsen.com/content/dam/nielsenglobal/vn/docs/Reports/2013/2013_VN_pocket_reference_book_low.pdf.

[bib56] NIN (2011). General nutrition survey.

[bib57] NIN (2012-2015). Dietary data from the national institute of nutrition Vietnam; accessible through bioversity international.

[bib59] Ortega D.L., Tschirley D.L. (2017). Demand for food safety in emerging and developing countries: A research agenda for Asia and sub-Saharan africa. Journal of Agribusiness in Developing and Emerging Economies.

[bib60] Páez A., Mercado R.G., Farber S., Morency C., Roorda M. (2010). Relative accessibility deprivation indicators for urban settings: Definitions and application to food deserts in Montreal. Urban Studies.

[bib61] Pingali P. (2007). Westernization of Asian diets and the transformation of food systems: Implications for research and policy. Food Policy.

[bib62] Poti J.M., Braga B., Qin B. (2017). Ultra-processed food intake and obesity: What really matters for health—processing or nutrient content?. Current Obesity Reports.

[bib63] Poti J., Mendez M., Ng S., Popkin B. (2015). Ultra-processed and ready-to-eat food and beverage purchases differ by race, education, and income in a longitudinal US study. The FASEB Journal.

[bib64] Pulliat G. (2013). Vulnérabilité alimentaire et trajectoires de sécurisation des moyens d’existence à Hanoi : Une lecture des pratiques quotidiennes dans une métropole émergente. Thèse de doctorat en géographie humaine et régionale.

[bib100] Raja S., Ma C., Yadav P. (2008). Beyond food deserts. Measuring and mapping racial disparities in neighbourhood food environments. Journal of Planning, Education and Research.

[bib66] Raneri J., Wertheim-Heck S. (2019). Retail diversity for dietary diversity: Resolving food-safety versus nutrition priorities in Hanoi. https://www.unscn.org/uploads/web/news/UNSCN-Nutrition44-WEB.pdf.

[bib67] Rauber F., Campagnolo P.D.B., Hoffman D.J., Vitolo M.R. (2015). Consumption of ultra-processed food products and its effects on children's lipid profiles: A longitudinal study. Nutrition, Metabolism, and Cardiovascular Diseases.

[bib68] Ravallion M., Shaohua C., Prem S. (2007). New evidence on the urbanization of global poverty. http://econ.worldbank.org/docsearch.

[bib69] Reardon T., Ruben R., Slingerland M., Nijhoff H. (2006). The rapid rise of supermarkets and the use of private standards in their food product procurement systems in developing countries. Agro-food chains and networks for development.

[bib70] Reardon T., Timmer C.P. (2012). The economics of the food system revolution. Annual Review of Resource Economics.

[bib71] Reardon T., Timmer C.P. (2014). Five inter-linked transformations in the Asian agrifood economy: Food security implications. Global Food Security.

[bib72] Reckwitz A. (2002). Toward a theory of social practices: A development in culturalist theorizing. European Journal of Social Theory.

[bib73] Samanta D., Heshmati A. (2015). Urban poverty in developing asia—dichotomy between the income and non-income dimensions: Are we not grossly underestimating its incidence?. Poverty reduction policies and practices in developing Asia, economic studies in inequality, social exclusion and well-being. Philippines.

[bib74] Schatzki T. (2016). Keeping track of large phenomena. Geografische Zeitschrift.

[bib75] Shepherd R., Towler G. (1992). Nutrition knowledge, attitudes and fat intake: Application of reasoned action. Journal of Human Nutrition and Dietetics.

[bib76] Shove E. (2010). Beyond the ABC: Climate change policy and theories of social change. Environment & Planning A.

[bib77] Shove E., Pantzar M., Watson M. (2012). The dynamics of social practice everyday life and how it changes.

[bib78] Spaargaren G., Lamers M., Weenink D. (2016). Practice Theory and Research. Exploring the dynamics of social life.

[bib79] Spronk I., Kullen C., Burdon C., O'Connor H. (2014). Relationship between nutrition knowledge and dietary intake. British Journal of Nutrition.

[bib80] Strengers Y., Moloney S., Maller C., Horne R., Strengers Y., Maller C. (2015). Beyond behaviour change: Practical applications of social practice theory in behaviour change programes. Social practices, interventions and sustainability.

[bib81] Taillie L.S., Ng S.W., Popkin B.M. (2015). Walmart and other food retail chains. Trends and disparities in the nutritional profile of packaged food purchases. American Journal of Preventive Medicine.

[bib82] Thang N.M., Popkin B.M. (2004). Patterns of food consumption in Vietnam: Effects on socioeconomic groups during an era of economic growth. European Journal of Clinical Nutrition.

[bib83] UN (2014). United nations world urbanization prospects:. http://esa.un.org/unpd/wup/.

[bib84] UN (2015). United nations world population prospects. The 2015 revision. http://esa.un.org/unpd/wpp/publications/files/key_findings_wpp_2015.pdf.

[bib85] Wang M., Kim S., Gonzalez A., MacLeod K., Winkleby M. (2007). Socioeconomic and food-related physical characteristics of the neighbourhood environment are associated with body mass index. Journal of Epidemiological Community Health.

[bib86] Warde A. (2016). The practice of eating.

[bib87] Warde A., Southerton D. (2012). The Habits of Consumption. Studies across disciplines in the humanities and social sciences.

[bib88] Welch D., Warde A., Reisch L.A., Thorgersen J. (2015). Theories of practice and sustainable consumption. Handbook of research on sustainable consumption.

[bib89] Wertheim-Heck S.C.O., Anh H.T.L., My H.T.T., Klaver M., Huong P.T.T. (2014). Reaching lower income groups with safe and healthy foods - mission possible? Insights into the consumption of lower income consumers in urban Hanoi. Fresh Studio. http://www.freshstudio.vn/images/media-archive/Publications/2014/20140612%20Reaching%20lower%20income%20groups%20with%20safe%20and%20healthy%20food%20-%20mission%20possible.pdf.

[bib90] Wertheim-Heck S., Raneri J.E., Oosterveer P. (2019). Food safety and nutrition for low-income urbanites: Exploring a social justice dilemma in consumption policy.

[bib91] Wertheim-Heck S.C.O., Spaargaren G. (2015). Shifting configurations of shopping practices and food safety dynamics in Hanoi, Vietnam; a historical analysis. Agriculture and Human Values.

[bib92] Wertheim-Heck S.C.O., Spaargaren G., Vellema S. (2014). Food safety in everyday life: Shopping for vegetables in a rural city in Vietnam. Journal of Rural Studies.

[bib93] Wertheim-Heck S.C.O., Vellema S., Spaargaren G. (2014). Constrained consumer practices and food safety concerns in Hanoi. International Journal of Consumer Studies.

[bib94] Wertheim-Heck S.C.O., Vellema S., Spaargaren G. (2015). Food safety and urban food markets in Vietnam: The need for flexible and customized retail modernization policies. Food Policy.

[bib95] Widener M.J.S.,J. (2014). When are food deserts? Integrating time into research on food accessibility. Health & Place.

[bib96] Wrigley N., Lowe M. (2010). The globalization of trade in retail services. Report commissioned by the OECD trade policy linkages and services division for the.

[bib97] Wrigley N., Warm D., Margetts B., Whelan A. (2002). Assessing the impact of improved retail access on diet in a ‘food desert’: A preliminary report. Urban Studies.

