# Supplementary material for: A cross-disciplinary mixed-method approach to understand how food retail environment transformations influence food choice and intake among the urban poor: Experiences from Vietnam
Source: Appetite. 2019 Nov 1;142:104370. doi: 10.1016/j.appet.2019.104370 (PMC6739597; doi:10.1016/j.appet.2019.104370)
Supplement: Multimedia component 1 [file mmc1.doc]

**SI 1 - Sampling Plan**

The below sampling strategy was the basis for (i) the household survey, (ii) the nutrition survey & 24hr diet recall, and (iii) the qualitative research. The strategy was divided into 4 steps, of which steps 1 to 3 were completed before the start of the survey fieldwork and of which the steps 3 and 4 were addressed during the field research

**STEP 1: poverty definition**

In this research a cut-off point for poverty of 5-5.5 USD/cap/day was used, building on the Vietnam Household Income Band (HIB)[[1]](#endnote-2),[[2]](#endnote-3) which follows the income classification used by the Vietnam General Statistics Office.[[3]](#endnote-4) These classifications are well understood by the local population and were used in previous research.[[4]](#endnote-5) For discussing the bottom of the pyramid internationally different cut-off points are used (from 1,25USD/cap/day (World Bank) to up to 8USD/cap/day.[[5]](#endnote-6) The world bank definition of poor at 1.25 USD/cap/day appears too narrow for an Asian urban context (see the table below). Given the price levels of basic necessities and high inflation rates in Hanoi and the urban context that does not allow for food self-provisioning (like in rural areas), we included respondents belonging to the D and C income strata, see table below. Depending on currency conversion rates this will be up to 5-5.5 USD/cap/day. In the below table the monthly household income band was recalculated into daily per capita income band based on an average household size of four people for urban Hanoi lower income groups, based on the official population census conducted in 2009. Further we used the currency conversion rate of 20 June 2016.

*Table 1*

| **HIB** | **Household income in VND/month** | **Per capita income in USD/day** | **% of urban population** |
| --- | --- | --- | --- |
| A/A1/A2+ | 15,000,000 -> | 5.5 -> | 39% |
| B | 7,500,000 - 14,499,999 | -5.5 | 41% |
| **C** | **4,500,000 - 7,499,999** | **1,67 – 5.5** | **33%** |
| **D** | **3,000,000 - 4,999,999** | **1.12 – 1.67** | **6%** |
| E | 1,500,000 - 2,999,999 | 0,56 – 1.12 | 0% |
| F | 0 - 1,499,999 | 0 – 0,56 | 0% |

Respondent inclusion was based on monthly household income and householdsize in order to allow inclusion based on per per capita income levels; see the respondent inclusion question, which was used in the household survey below:

*Table 2*

| **Q8** | **A** | **How many persons are living with you within your household? [SA]** | | | | | | | | |
| --- | --- | --- | --- | --- | --- | --- | --- | --- | --- | --- |
|  |  |  | 2 pers | 3 pers | 4 pers | 5 pers | 6 pers | 7 pers | 8 pers | 8+ pers |
| **B** |  | Code | 1 | 2 | 3 | 4 | 5 | 6 | 7 | 8 |
| **What is your households monthly income** | Less than 3 mln vnd | 1 | Continue | | | | | | | |
|  | 3,000,000 - 4,499,999 | 2 |  |  |  |  |  |  |  |  |
|  | 4,500,000-5,499,999 | 3 |  |  |  |  |  |  |  |  |
|  | 5,500,000-6,499,999 | 4 |  |  |  |  |  |  |  |  |
|  | 6,500,000-7,499,999 | 5 | STOP | Continue | | | | | | |
|  | 7,500,000-8,499,999 | 6 | STOP |  |  |  |  |  |  |  |
|  | 8,500,000-9,499,999 | 7 | STOP |  |  |  |  |  |  |  |
|  | 9,500,000-10,499,999 | 8 | STOP |  |  |  |  |  |  |  |
|  | 10,500,000-11,499,999 | 9 | STOP | STOP | Continue | | | | | |
|  | 11,500,000-12,499,999 | 10 | STOP | STOP |  |  |  |  |  |  |
|  | 12,500,000-13,499,999 | 11 | STOP | STOP |  |  |  |  |  |  |
|  | 13,500,000-14,999,999 | 12 | STOP | STOP | STOP | Continue | | | | |
|  | 15mln and over | 13 | STOP | | | | | | | |

**STEP 2: district selection**

Districts are purposively selected based on below criteria:

1. Exclusion of recently developed urban areas on the outer-skirts of urban Hanoi (like Ha Dong)
2. Focus on the more inner urban districts
3. Exclusion of a-typical districts: Tay Ho (expat community) and Hoan Kiem (tourist area)
4. Final district selection based on:

- Retail density (supermarket)
- Information readily available on markets (Health Bridge)
- Size in square km (feasibility for census)
- Percentage poor population according to GSO statistics (although we do not focus on the official poor living below 1usd/cap/day)

Out of the 5 relevant urban districts two districts have been selected: Dong Da and Ba Dinh.

- Dong Da has the highest population density, the highest number of supermarkets.
- Ba Dinh is a very central district (Old Hanoi), with the highest rate of poor household

*Table 3*

| **District** | **No. Wards** | **Size (km2)** | **Population** | **Density** | **Poverty rates (less than 1USD/cap/day** | **Supermarkets** |
| --- | --- | --- | --- | --- | --- | --- |
| **Ba Dinh** | **14** | **9.224** | **242800** | **26323** | **0.92%** | **10** |
| Cau Giay | 8 | 12.04 | 251800 | 20914 | 0.12% | 10 |
| **Dong Da** | **21** | **9.96** | **408000** | **40964** | **0.55%** | **15** |
| Hai Ba Trung | 20 | 14.6 | 378000 | 25890 | 0.66% | 9 |
| Thanh Xuan | 11 | 9.11 | 259000 | 28430 | 0.25% | 5 |

*Note: Selected districts in bold print*

- *Household Living Standard Survey; General Statistics Office Of Vietnam; 2012*
- *Statistics of Hanoi population & labour, Hanoi Statistics Office, 2013*

*Figure 1*


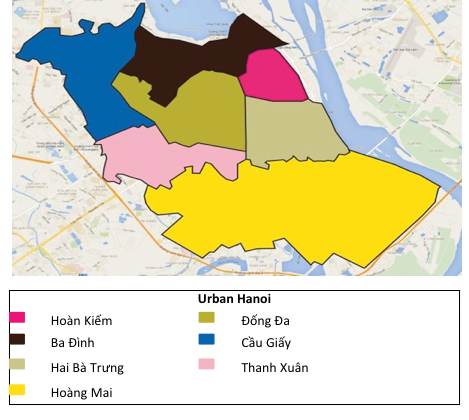


**STEP 3: street selection**

In the districts Dong Da and Ba Dinh, a census of the retail outlets, as per the project proposal, has been conducted. The census data provide the basis for survey area, read street, selection for conducting the household survey. Based on the GIS census mapping concentric circles were drawn around the identified hyper-and supermarkets and formal wet markets to identify areas within the four sampling strata shown in the figure 2 and table 4 below:

*Table 4*

| **Respondents** | **Supermarket within walking distance (300m)** | **Formal wet-market within walking distance (300m)** |
| --- | --- | --- |
| Group 1 (N=100) | Yes | Yes |
| Group 2 (N=100) | Yes | No |
| Group 3 (N=100) | No | Yes |
| Group 4 (N=100) | No | No |

*Figure 2*

The enumerators were assigned to a specific area. The enumerators were devided over the two districts, but the district were not be a variable in our analysis. A balanced distribution between the two districts was prefered, but because the district was not a variable in our data-analysis, it was not a serious problem that for practical reasons during the excution, slightly more or less households are included per group in one district compared to the other, as long as the total numers per strata were met.

*Table 5*

|  | **Respondents** | **Supermarket within walking distance (300m)** | **Formal wet-market within walking distance (300m)** |
| --- | --- | --- | --- |
| **Ba Dinh** | Group 1 (N=50) | Yes | Yes |
|  | Group 2 (N=50) | Yes | No |
|  | Group 3 (N=50) | No | Yes |
|  | Group 4 (N=50) | No | No |
| **Dong Da** | Group 1 (N=50) | Yes | Yes |
|  | Group 2 (N=50) | Yes | No |
|  | Group 3 (N=50) | No | Yes |
|  | Group 4 (N=50) | No | No |

**STEP 4: Household/respondent selection**

The survey was a door to door survey in which participants were randomly selected within the aformentioned areas. The enumerator assigned to a specific area randomly selected a street to start the fieldwork; went to a random house or apartment building within the street and approach respondents:

- Within one and the same apartment building no more than 3 households/apartments were included.
- Only included every 3rd apartment within the apartment building.
- Only included every 3rd building on the street
- Further paticipant inclusion criteria were included in the survey questionnaire.

**Proceedings in short:**

- 1. Completed survey: continue to the 3rd next apartment within the building )(max of 3 inclusions per apartment building), or to the 3rd next building.
  2. Unqualified survey: continue to the next apartment within the building or to the next building.

**STEP 5: Time slots**

To avoid a bias based on execution timing during the day, the enumerators operated in 3 different time slots:

*Table 6*

| **Period** | **Timeslot*** | **Rationale** |
| --- | --- | --- |
| Morning | 7-11am | Previous research has indicated that most people are shopping in the early morning, this timeslot thus risks to exclude morning shoppers. |
| Afternoon | 2-6pm | The afternoon allows for the inclusion of people without a job, who are mostly shopping in the morning and using the cooler evening hours for other activities |
| Evening | 6-10pm | PREFERENCE:  Most people are expected to be at home and it is further expected that women responsible for household shopping and cooking will have more time available in the evening than during daytime |

**Lunch time was excluded*

1. Nielsen (2013) Know Your Consumers Grow Your Business 2013 Pocket Reference Book Vietnam.  [↑](#endnote-ref-2)
2. Nielsen (2014). Know Act Grow. Driving smarter business decisions in Vietnam. Available online: http://www.nielsen.com/content/dam/nielsenglobal/vn/docs/Reports/2014/Know-Act-Grow-nov-2014.pdf [↑](#endnote-ref-3)
3. GSO General Statistics Office Vietnam (2010) Results of the Vietnam Household Living Standard Survey 2010; http://www.gso.gov.vn [↑](#endnote-ref-4)
4. Wertheim-Heck et al. (2014) Reaching lower income groups with safe and healthy foods – mission possible? Insights into the consumption of lower income consumers in urban Hanoi. Fresh Studio Report. Available online: http://www.freshstudio.vn/images/media-archive/Publications/2014/20140612%20Reaching%20lower%20income%20groups%20with%20safe%20and%20healthy%20food%20-%20mission%20possible.pdf [↑](#endnote-ref-5)
5. BoP Innovation Center (2013) BoP Innovation Cycle: The process of innovation to create inclusive business. [↑](#endnote-ref-6)
