# Supplementary material for: A cross-disciplinary mixed-method approach to understand how food retail environment transformations influence food choice and intake among the urban poor: Experiences from Vietnam
Source: Appetite. 2019 Nov 1;142:104370. doi: 10.1016/j.appet.2019.104370 (PMC6739597; doi:10.1016/j.appet.2019.104370)
Supplement: Multimedia component 2 [file mmc2.docx]

**SI 3 – Household survey questionnaire**

| QC No: | | | Interviewer No: | |
| --- | --- | --- | --- | --- |
| Location/district:  Ba Dinh ………………………1  Dong Da ..……………………2 | | | Day…………………….Month…………….. Year 2017  Monday …………………………………………1  Tuesday …………………………………………2  Wednesday ……………………………………3  Thursday ……………………………………….4  Friday …………………………………………….5  Saturday ………………………………………..6  Sunday …………………………………………..7 | |
| Respondent Name: | | | Respondent phone No.: | |
| Address: | GPS coordinates | No | Alley | Street/road |
| Questionnaire starting time: | | | Questionnaire finishing time: | |
|  | | |  | |

| INTERVIEWER’S STATEMENT  I have finished the questionnaire according to the instructions of the project  (Signature and name in full) | SUPERVISOR’S STATEMENT  I have checked this questionnaire according to the instructions of the project  (Signature and name in full) |
| --- | --- |

[SA] = Single Answer only | [MA] = Multiple Answers possible/allowed |[OA] = Open Answer

**Include WOMEN only**

**Include only the respondents that are willing to participate in both this survey AND the nutrition survey**

| Part 1- Screening |
| --- |

| Q1 | Please answer the following question.  Who is the main person responsible for the food shopping in your household? [SA] | Code | Route |
| --- | --- | --- | --- |
|  | I am | 1 | Continue |
|  | Other person | 2 | Ask to talk to this other person if not possible or if not female stop |

| Q2 | Please note down the gender of the respondent [SA] | Code | Route |
| --- | --- | --- | --- |
|  | Female | 1 | Continue |
|  | Male | 2 | Stop |

| Q3 | Would you be willing to also participate in an additional research about the foods consumed in your household? [for explanation please refer to the informed consent information above] | Code | Route |
| --- | --- | --- | --- |
|  | Yes | 1 | Continue |
|  | No | 2 | Stop |

| Q4 | Who does the food preparation/cooking in your household? [SA] | Code | Route |
| --- | --- | --- | --- |
|  | **I am the** **ONLY** person in charge of deciding on the menu of the day, the preparation of the ingredients and the cooking of the dishes. | 1 |  |
|  | There is no clear division of labor. We are all in charge of deciding on the menu of the day, the preparation of the ingredients and the cooking of the dishes. | 2 | Continue |
|  | Someone else / some others are in charge of deciding on the menu of the day, the preparation of the ingredients and the cooking of the dishes. | 3 | Stop |

| Q5 | What is your year of birth? [OA] |
| --- | --- |
|  | …………………………….……………………………………………………………………………………… |

|  | During or after 1967 | 1 | Continue |
| --- | --- | --- | --- |
|  | Before 1967 | 2 | Stop |

| Q6 | How long have you been living here at this address? [SA] | Code | Route |
| --- | --- | --- | --- |
|  | Less than 2 years | 1 | Stop |
|  | 2 years or more | 2 | Continue |

| Q7 | What is your household size? [SA] | Code | Route |
| --- | --- | --- | --- |
|  | Single household | 1 | Stop |
|  | 2 or more people | 2 | Continue |

| Q8 | A | How many persons are living with you within your household? [SA] | | | | | | | | |
| --- | --- | --- | --- | --- | --- | --- | --- | --- | --- | --- |
|  |  |  | 2 pers | 3 pers | 4 pers | 5 pers | 6 pers | 7 pers | 8 pers | 8+ pers |
| B |  | Code | 1 | 2 | 3 | 4 | 5 | 6 | 7 | 8 |
| What is your households monthly income | Less than 3 mln vnd | 1 | Continue | | | | | | | |
|  | 3,000,000 - 4,499,999 | 2 |  |  |  |  |  |  |  |  |
|  | 4,500,000-5,499,999 | 3 |  |  |  |  |  |  |  |  |
|  | 5,500,000-6,499,999 | 4 |  |  |  |  |  |  |  |  |
|  | 6,500,000-7,499,999 | 5 | STOP | Continue | | | | | | |
|  | 7,500,000-8,499,999 | 6 | STOP |  |  |  |  |  |  |  |
|  | 8,500,000-9,499,999 | 7 | STOP |  |  |  |  |  |  |  |
|  | 9,500,000-10,499,999 | 8 | STOP |  |  |  |  |  |  |  |
|  | 10,500,000-11,499,999 | 9 | STOP | STOP | Continue | | | | | |
|  | 11,500,000-12,499,999 | 10 | STOP | STOP |  |  |  |  |  |  |
|  | 12,500,000-13,499,999 | 11 | STOP | STOP |  |  |  |  |  |  |
|  | 13,500,000-14,999,999 | 12 | STOP | STOP | STOP | Continue | | | | |
|  | 15mln and over | 13 | STOP | | | | | | | |

| Part 2- Food shopping PRACTICES |
| --- |

| Q9 | How many times per week do you/your family buy food for your household? [SA] | Code |
| --- | --- | --- |
|  | Every day | 1 |
|  | 4-6 times a week | 2 |
|  | 2 to 3 times a week | 3 |
|  | Once a week | 4 |

| Q10 | How often do you buy your food at these places? [SA – Showcard outlets] | | | | | | |
| --- | --- | --- | --- | --- | --- | --- | --- |
|  |  | Hypermarket/ Supermarket | Formal wet market | Informal street market | Convenience store/ Minimart | Specialty store | Street vendor |
|  | Every day | 1 | 1 | 1 | 1 | 1 | 1 |
|  | 4-6 times a week | 2 | 2 | 2 | 2 | 2 | 2 |
|  | 2-3 times a week | 3 | 3 | 3 | 3 | 3 | 3 |
|  | Once a week | 4 | 4 | 4 | 4 | 4 | 4 |
|  | < once a week | 5 | 5 | 5 | 5 | 5 | 5 |
|  | Never | 6 | 6 | 6 | 6 | 6 | 6 |

| Q11 | Could you please tell me the name of the supermarket and/or official wet market you where you shop at least once a week? [OA]  If no hyper/supermarket or wet market is visited once per week in Q10, please continue with Q12 | | |
| --- | --- | --- | --- |
|  |  | Code | Name of the place/address |
|  | Hypermarket/Supermarket | 1 |  |
|  | Formal Wet market | 2 |  |

| Q12 | At what moment of the day do you normally shop? [SA] | Code |
| --- | --- | --- |
|  | Early morning: before 8:00 | 1 |
|  | Morning: 8:00-11:00 | 2 |
|  | Lunch: 11:00-13:00 | 3 |
|  | Afternoon: 13:00-16:30 | 4 |
|  | Late afternoon: 16:30-19:00 | 5 |
|  | Evening: after 19:00 | 6 |

| Q13 | Where (what type of outlet) do you/your household buy most (QUANTITY) of the foods your household consumes during a week? [SA – Showcard outlets] | Code |
| --- | --- | --- |
|  | Hypermarket/ Supermarket | 1 |
|  | Formal wet market | 2 |
|  | Informal street market | 3 |
|  | Convenience store/ Minimart | 4 |
|  | Specialty store (e.g. safe vegetable shop, fruits shop, meat shop,…) | 5 |
|  | Street vendor | 6 |

| Q14 | How much do you agree with the following statements? [SA per statement] | | | | | |
| --- | --- | --- | --- | --- | --- | --- |
|  | “**The reason I buy most food from** the shop I buy most of my foods (place is the highest ranking answer Q10a) **is because (of)**…” | Totally disagree | Disagree | Neutral | Agree | Totally agree |
| A | I am used to shop here; it’s a habit | 1 | 2 | 3 | 4 | 5 |
| B | Lowest selling price | 1 | 2 | 3 | 4 | 5 |
| C | It is close to my home | 1 | 2 | 3 | 4 | 5 |
| D | It is located on the way from home to work/school/… | 1 | 2 | 3 | 4 | 5 |
| E | The foods offered are safe for consumption | 1 | 2 | 3 | 4 | 5 |
| F | I enjoy shopping here | 1 | 2 | 3 | 4 | 5 |
| G | Wide assortment offered | 1 | 2 | 3 | 4 | 5 |
| H | They offer healthy foods | 1 | 2 | 3 | 4 | 5 |
| I | The personal contact with the vendor | 1 | 2 | 3 | 4 | 5 |

| Q15 | What means of transportation do you use for shopping foods for your household? [MA possible and please indicate the frequency of transportation means] | | | | | |
| --- | --- | --- | --- | --- | --- | --- |
|  |  | Never | Seldom | Sometimes | Most of the times | Always |
| A | None, I walk | 1 | 2 | 3 | 4 | 5 |
| B | Bicycle | 1 | 2 | 3 | 4 | 5 |
| C | Motorbike | 1 | 2 | 3 | 4 | 5 |
| D | Car | 1 | 2 | 3 | 4 | 5 |

| Part 3- Food shopping PREFERENCES |
| --- |

| Q16 | How many times per week do you/your family PREFER to shop for food for your household? [SA] | Code |
| --- | --- | --- |
|  | Every day | 1 |
|  | 4-6 times a week | 2 |
|  | 2 to 3 times a week | 3 |
|  | Once a week | 4 |

| Q17 | At what moment of the day would you PREFER to shop? [SA] | Code |
| --- | --- | --- |
|  | Early morning, before 8:00 | 1 |
|  | Morning between 8:00-11:00 | 2 |
|  | Lunch: between 11:00-13:00 | 3 |
|  | Afternoon between 13:00-16:30 | 4 |
|  | Late afternoon: 16:30-19:00 | 5 |
|  | Evening: after 19:00 | 6 |

| Q18 | Where (what type of outlet) would you PREFER to buy food for your household? [SA – Showcard outlets] | Code |
| --- | --- | --- |
|  | Hypermarket/ Supermarket | 1 |
|  | Official wet market | 2 |
|  | Informal street market | 3 |
|  | Convenience store/ Minimart | 4 |
|  | Specialty store | 5 |
|  | Street vendor | 6 |

| Q19 | How much do you agree with the following statements? [SA per statement] | | | | | |
| --- | --- | --- | --- | --- | --- | --- |
|  |  | Totally disagree | Disagree | Neutral | Agree | Totally agree |
| A | Buying food in supermarkets is too time consuming | 1 | 2 | 3 | 4 | 5 |
| B | Buying food in supermarkets is interesting | 1 | 2 | 3 | 4 | 5 |
| C | Buying food in supermarkets is desirable | 1 | 2 | 3 | 4 | 5 |
| D | Buying food in supermarkets is expensive | 1 | 2 | 3 | 4 | 5 |
| E | Foods offered in supermarkets are safe for consumption | 1 | 2 | 3 | 4 | 5 |
| F | If I could I would only buy at supermarkets | 1 | 2 | 3 | 4 | 5 |
| G | Buying food at wet markets is too time consuming | 1 | 2 | 3 | 4 | 5 |
| H | Buying food at wet markets is interesting | 1 | 2 | 3 | 4 | 5 |
| I | Buying food at wet markets is desirable | 1 | 2 | 3 | 4 | 5 |
| J | Buying food at wet markets is expensive | 1 | 2 | 3 | 4 | 5 |
| K | Foods offered at wet markets are safe for consumption | 1 | 2 | 3 | 4 | 5 |
| L | If I could I would only buy at wet markets | 1 | 2 | 3 | 4 | 5 |

| Part 4- Food selection |
| --- |

| Q20 | What is the average daily food budget of your household? [SA] | Code |
| --- | --- | --- |
|  | Less than 50,000 VND | 1 |
|  | 50,000- 99,999 VND | 2 |
|  | 100,000-199,999 VND | 3 |
|  | 200,000VND-299,999 VND | 4 |
|  | 300,000-400,000 VND | 5 |
|  | More than 400,000 VND | 6 |

| Q21 | Where do you usually buy the following food items? [SA – Showcard outlet types]] | | | | | | |
| --- | --- | --- | --- | --- | --- | --- | --- |
|  |  | Hyper-/ Supermarket | Formal wet market | Informal Street market | Convenience store | Specialty store | Street vendor |
| A | Rice, cereals and starch products | 1 | 2 | 3 | 4 | 5 | 6 |
| B | Fish and Seafood | 1 | 2 | 3 | 4 | 5 | 6 |
| C | Meat, chicken (including organ) | 1 | 2 | 3 | 4 | 5 | 6 |
| D | Eggs | 1 | 2 | 3 | 4 | 5 | 6 |
| E | Dark green leafy vegetables | 1 | 2 | 3 | 4 | 5 | 6 |
| F | Roots and tubers | 1 | 2 | 3 | 4 | 5 | 6 |
| G | Orange vegetables (pumpkin, sweet potato, carrot, red bell pepper) | 1 | 2 | 3 | 4 | 5 | 6 |
| H | Orange flesh fruits (ripe papaya, ripe mango, persimmon, passionfruit, orange muskmelon/rockmelon, dried apricots or peaches, NOT: Orange fruit (qua cam)) | 1 | 2 | 3 | 4 | 5 | 6 |
| I | Other fruits and vegetables | 1 | 2 | 3 | 4 | 5 | 6 |
| J | Legumes | 1 | 2 | 3 | 4 | 5 | 6 |
| K | Dairy products | 1 | 2 | 3 | 4 | 5 | 6 |
| L | Snack foods, ultra-processed foods (e.g. instant noodles) | 1 | 2 | 3 | 4 | 5 | 6 |
| M | Spices, condiments, beverages | 1 | 2 | 3 | 4 | 5 | 6 |
| N | Oils and fats | 1 | 2 | 3 | 4 | 5 | 6 |
| O | Nuts and seeds | 1 | 2 | 3 | 4 | 5 | 6 |
| P | Insects | 1 | 2 | 3 | 4 | 5 | 6 |
| Q | Sweets | 1 | 2 | 3 | 4 | 5 | 6 |

| Q22 | Which food items would you prefer to buy **more** if your food budget increases? [Top 3] | Code |
| --- | --- | --- |
|  | Rice, cereals and starch products | 1 |
|  | Fish and Seafood | 2 |
|  | Meat, chicken (including organ) | 3 |
|  | Eggs | 4 |
|  | Dark green leafy vegetables | 5 |
|  | Roots and tubers | 6 |
|  | Orange vegetables (pumpkin, sweet potato, carrot) | 7 |
|  | Orange flesh fruits (papaya, mango) | 8 |
|  | Other fruits and vegetables | 9 |
|  | Legumes | 10 |
|  | Dairy products | 11 |
|  | Snack foods, ultra-processed foods (e.g. instant noodles) | 12 |
|  | Spices, condiments, beverages | 13 |
|  | Oils and fats | 14 |
|  | Nuts and seeds | 15 |
|  | Insects | 16 |
|  | Sweets | 17 |

| Q23 | How important are the following factors to you when you decide what vegetables to buy? [SA] | | | | | |  |
| --- | --- | --- | --- | --- | --- | --- | --- |
|  |  | Totally unimportant | Unimportant | Neutral | Important | Absolutely important |  |
| A | Taste preference of me and my family members | 1 | 2 | 3 | 4 | 5 |  |
| C | Food safety | 1 | 2 | 3 | 4 | 5 |  |
| B | Health (nutritional value) | 1 | 2 | 3 | 4 | 5 |  |
| D | Easy to prepare | 1 | 2 | 3 | 4 | 5 |  |
| E | Low price | 1 | 2 | 3 | 4 | 5 | |

| Q24 | Please finish the following sentence and indicate the importance of the complete statement.  *'It is important to me that the food I eat on a typical day..…'* | | | | | |
| --- | --- | --- | --- | --- | --- | --- |
|  |  | Totally unimportant | Unimportant | Neutral | Important | Absolutely important |
| A | Contains a lot of vitamins and minerals | 1 | 2 | 3 | 4 | 5 |
| B | Keeps me healthy | 1 | 2 | 3 | 4 | 5 |
| C | Is nutritious | 1 | 2 | 3 | 4 | 5 |
| D | Is high in protein | 1 | 2 | 3 | 4 | 5 |
| E | Is easy to clean | 1 | 2 | 3 | 4 | 5 |
| F | Can be prepared easily | 1 | 2 | 3 | 4 | 5 |
| G | Can be bought close to where I live or work | 1 | 2 | 3 | 4 | 5 |
| H | Is easily available | 1 | 2 | 3 | 4 | 5 |
| I | Is affordable | 1 | 2 | 3 | 4 | 5 |
| J | Is cheap | 1 | 2 | 3 | 4 | 5 |
| K | Is good value for money | 1 | 2 | 3 | 4 | 5 |

| Part 5 – VEGETABLES - Food safety RISK perception |
| --- |

| Q25 | How much do you agree with the below statement regarding your concern with the safety of the vegetables you buy for your household? [SA] | | | | | |
| --- | --- | --- | --- | --- | --- | --- |
|  |  | Totally not agree | Not agree | Neutral | Agree | Totally Agree |
| A | I am ALWAYS concerned (frequency) | 1 | 2 | 3 | 4 | 5 |
| B | I am VERY MUCH concerned (severity) | 1 | 2 | 3 | 4 | 5 |

| Q26 | How concerned are you about the following sources of food safety risks[SA] | | | | | |
| --- | --- | --- | --- | --- | --- | --- |
|  |  | Totally not concerned | Not so concerned | Neutral | Rather concerned | Totally concerned |
| A | Fertilizers and growth enhancers | 1 | 2 | 3 | 4 | 5 |
| B | Pesticides | 1 | 2 | 3 | 4 | 5 |
| C | Preservation treatment | 1 | 2 | 3 | 4 | 5 |
| D | Use of /growing with contaminated water and soil | 1 | 2 | 3 | 4 | 5 |
| E | Unhygienic handling practices (farm to store) | 1 | 2 | 3 | 4 | 5 |

| Q27 | How much do you agree with the following statements? | | | | | |
| --- | --- | --- | --- | --- | --- | --- |
|  |  | Totally disagree | Disagree | Neutral | Agree | Totally Agree |
| A | I only select vegetables that have a peel | 1 | 2 | 3 | 4 | 5 |
| B | I only select vegetables which look fresh/green | 1 | 2 | 3 | 4 | 5 |
| C | I only select vegetables which are a bit eaten by worms | 1 | 2 | 3 | 4 | 5 |
| D | I only select vegetables that are on season | 1 | 2 | 3 | 4 | 5 |
| E | I only select vegetables that carry food safety certification | 1 | 2 | 3 | 4 | 5 |
| F | I only buy vegetables when I know where they are produced | 1 | 2 | 3 | 4 | 5 |
| G | I buy what my vendor advices me to buy | 1 | 2 | 3 | 4 | 5 |

| Q28 | How much do you agree with the following statements? [SA] | | | | | |
| --- | --- | --- | --- | --- | --- | --- |
|  |  | Totally disagree | Disagree | Neutral | Agree | Totally agree |
| A | I am less concerned about the safety of vegetables when they are in-season than off-season. | 1 | 2 | 3 | 4 | 5 |
| B | Vegetables offered in supermarkets are safer than in wet markets. | 1 | 2 | 3 | 4 | 5 |
| C | Vegetables offered in wet markets are safer than in street markets/vendors | 1 | 2 | 3 | 4 | 5 |
| D | It is safer to consume vegetables that are certified safe than uncertified vegetables. | 1 | 2 | 3 | 4 | 5 |
| E | Vegetables offered in wet markets are safer than in supermarkets | 1 | 2 | 3 | 4 | 5 |
| F | Vegetables offered in street markets/vendors are safer than in wet markets | 1 | 2 | 3 | 4 | 5 |
| G | I feel more safe when I buy from a vendor I trust, than when I buy certified vegetables | 1 | 2 | 3 | 4 | 5 |
| H | I am concerned about safety but make most purchasing decisions based on price | 1 | 2 | 3 | 4 | 5 |

| Q29 | If you are shopping for vegetables, but are worried about food safety in the retail outlet, what do you do? [SA]  Retail outlet refers to the place where you are shopping for vegetables; [Showcard of retail types can be used if unclear to the respondent] | Code |
| --- | --- | --- |
|  | Buy the same food and quantity anyway | 1 |
|  | Buy the same food but a lesser quantity | 2 |
|  | Buy different/less preferred food | 3 |
|  | Change retail outlet | 4 |

| Q30 | How often do you feel confident in protecting yourself and your household members from food safety risks? [SA] | Code |
| --- | --- | --- |
|  | Yes, always | 1 |
|  | Yes, sometimes | 2 |
|  | Neutral | 3 |
|  | Seldom | 4 |
|  | Never | 5 |

| Q31 | Independently from the source, can you rate how you generally consider the following vegetables in terms of safety? (1 would mean “Not safe at all” and 5 would mean “Very safe” with each statement) | | | | | |
| --- | --- | --- | --- | --- | --- | --- |
|  |  | Not safe at all | Risky | Neutral | Rather safe | Very safe |
| A | Roots and tubers | 1 | 2 | 3 | 4 | 5 |
| B | Squash, gourds, pumpkin | 1 | 2 | 3 | 4 | 5 |
| C | Dark green leafy vegetables | 1 | 2 | 3 | 4 | 5 |
| E | Orange sweet potato | 1 | 2 | 3 | 4 | 5 |
| F | Carrots | 1 | 2 | 3 | 4 | 5 |
| G | Beans and legumes | 1 | 2 | 3 | 4 | 5 |
| H | Tomato | 1 | 2 | 3 | 4 | 5 |
| I | Red capsicum | 1 | 2 | 3 | 4 | 5 |
| J | Green beans | 1 | 2 | 3 | 4 | 5 |
| K | Cabbage | 1 | 2 | 3 | 4 | 5 |
| L | Mushrooms | 1 | 2 | 3 | 4 | 5 |
| M | Kang kong/ morning glory | 1 | 2 | 3 | 4 | 5 |
| N | Light green lettuce | 1 | 2 | 3 | 4 | 5 |
| O | Cauliflower, broccoli | 1 | 2 | 3 | 4 | 5 |
| P | Pak choy |  |  |  |  |  |
| Q | Zucchini | 1 | 2 | 3 | 4 | 5 |
| R | Eggplant | 1 | 2 | 3 | 4 | 5 |

| Part 6 – VEGETABLES – TRUST in Food Safety |
| --- |

| Q32 | To what extent do you believe that the vegetables sold under below mentioned claims are safe? [Showcard food safety claim indicators] | | | | | |
| --- | --- | --- | --- | --- | --- | --- |
|  |  | I totally NOT believe | I do not believe | neutral | I moderately believe | I totally believe |
| A | VietGAP certification | 1 | 2 | 3 | 4 | 5 |
| B | Rau an toan (safe vegetables) | 1 | 2 | 3 | 4 | 5 |
| C | Rau sach (clean vegetables) | 1 | 2 | 3 | 4 | 5 |
| D | Rau Huu Co (organic vegetables) | 1 | 2 | 3 | 4 | 5 |
| E | Private label (retail brand) |  |  |  |  |  |
| F | Producer brand |  |  |  |  |  |

| Q33 | What makes you trust the safety of the vegetables the most? [SA] | Code |
| --- | --- | --- |
|  | Your own judgement based on the external appearance of vegetables | 1 |
|  | Certificate given by authority attesting that the vegetable is safe | 2 |
|  | Retailer branding | 3  3 |
|  | Advice from regular vendor about food safety | 4 |
|  | Advice from relatives/friends/neighbours | 5 |
|  | Information on producer and production area | 6 |
|  | Others, please specify………. | 7 |

| Q34 | Do you think the way you clean vegetables is enough to make them safe for consumption? | Code |
| --- | --- | --- |
|  | Yes, totally | 1 |
|  | Yes, partly | 2 |
|  | No, not at all | 3 |

| Q35 | Have you or your family ever been sick due to consumption of vegetables? | Code |
| --- | --- | --- |
|  | Yes | 1 |
|  | No | 2 |

| Q36 | The following statements are about problems that you might face when buying **SAFE** food.  How much do you agree with the following statement? (Single code) | | | | | |
| --- | --- | --- | --- | --- | --- | --- |
|  | **“When I want to buy SAFE food,.…”** | Totally disagree | Disagree | Neutral | Agree | Totally agree |
| A | the price is too high. | 1 | 2 | 3 | 4 | 5 |
| B | they are often not available. | 1 | 2 | 3 | 4 | 5 |
| C | they are often not fresh. | 1 | 2 | 3 | 4 | 5 |
| D | the assortment is limited. | 1 | 2 | 3 | 4 | 5 |
| E | I don’t trust the food safety claim | 1 | 2 | 3 | 4 | 5 |
| F | there are no problems for me. | 1 | 2 | 3 | 4 | 5 |

| Part 7 – Respondent’s information |
| --- |

| Q37 | What is your highest education level? [SA] | Code |
| --- | --- | --- |
|  | No formal education | 1 |
|  | Primary school | 2 |
|  | Secondary school | 3 |
|  | High school | 4 |
|  | Vocational school | 5 |
|  | Undergraduate | 6 |
|  | Postgraduate | 7 |

| Q38 | Do you care for …. within your household? [MA possible] | | |
| --- | --- | --- | --- |
|  |  | Code | |
|  |  | Yes | No |
| A | Children 0-4 yrs | 1 | 2 |
| B | Children 5-12yrs | 1 | 2 |
| C | Elderly people (60yrs and older) | 1 | 2 |
| D | People that are ill | 1 | 2 |

| Q39 | Is a woman from an older generation living with you within the household, who is or was responsible for food shopping? [SA] | Code | Route |
| --- | --- | --- | --- |
|  | Yes | 1 | Continue with Q40 |
|  | NO | 2 | STOP |

| Q40 | Would you be willing to participate in a follow-up more in-depth research later this year/next year, which includes a multi- generation household interview? [SA] | Code |
| --- | --- | --- |
|  | Yes | 1 |
|  | No | 2 |

| END |
| --- |
